# Do monkeys compare themselves to others?

**DOI:** 10.1007/s10071-015-0943-4

**Published:** 2015-11-28

**Authors:** Vanessa Schmitt, Ira Federspiel, Johanna Eckert, Stefanie Keupp, Laura Tschernek, Lauriane Faraut, Richard Schuster, Corinna Michels, Holger Sennhenn-Reulen, Thomas Bugnyar, Thomas Mussweiler, Julia Fischer

**Affiliations:** Social Cognition Center Cologne, University of Cologne, Richard-Strauss-Str. 2, 50931 Cologne, Germany; Cognitive Ethology Laboratory, German Primate Center, Kellnerweg 4, 37077 Göttingen, Germany; Department of Cognitive Biology, University of Vienna, Althanstrasse 14, 1090 Vienna, Austria; Department of Forest and Conservation Sciences, University of British Columbia, Vancouver, BC V6T 1Z4 Canada; Leibniz ScienceCampus Primate Cognition, Kellnerweg 4, 37077 Göttingen, Germany

**Keywords:** Co-acting paradigm, Evolution, Inequity aversion, Meta-cognition, Monkeys, Non-human primates, Social comparison processes, Social relationships

## Abstract

Social comparisons are a fundamental characteristic of human behaviour, yet relatively little is known about their evolutionary foundations. Adapting the co-acting paradigm from human research (Seta in J Pers Soc Psychol 42:281–291, 1982. doi:10.1037//0022-3514.42.2.281), we examined how the performance of a partner influenced subjects’ performance in long-tailed macaques (*Macaca fascicularis*). Using parallel testing in touch screen setups in which subjects had to discriminate familiar and novel photographs of men and women, we investigated whether accuracy and reaction time were influenced by partner performance and relationship quality (affiliate vs. non-affiliate). Auditory feedback about the alleged performance of the co-actor was provided via playback; partner performance was either moderately or extremely better or worse than subject performance. We predicted that subjects would assimilate to moderately different comparison standards as well as to affiliates and contrast away from extreme standards and non-affiliates. Subjects instantly generalized to novel pictures. While accuracy was not affected by any of the factors, long reaction times occurred more frequently when subjects were tested with a non-affiliate who was performing worse, compared to one who was doing better than them (80 % quantile worse: 5.1, better: 4.3 s). For affiliate co-actors, there was no marked effect (worse: 4.4, better: 4.6 s). In a control condition with no auditory feedback, subjects performed somewhat better in the presence of affiliates (*M* = 77.8 % correct) compared to non-affiliates (*M* = 71.1 %), while reaction time was not affected. Apparently, subjects were sensitive to partner identity and performance, yet variation in motivation rather than assimilation and contrast effects may account for the observed effects.

**Electronic supplementary material:**

The online version of this article (doi:10.1007/s10071-015-0943-4) contains supplementary material, which is available to authorized users.

## Introduction

Comparing oneself to others is a fundamental element of human behaviour (Festinger 1954). We compare ourselves to others when we evaluate our abilities, performance (Mussweiler et al. 2004a), or attractiveness (Brown et al. 1992; for a review see Mussweiler 2003). Importantly, social comparisons are so pervasive that even fleeting exposure to a comparison standard may influence peoples’ self-evaluations (Mussweiler et al. 2004b). Because of the ubiquity and importance of social comparisons in humans, several studies have investigated the psychological mechanisms and processes underpinning social comparisons in humans (Festinger 1954; Mussweiler 2003; Corcoran et al. 2011). This research revealed that a variety of factors influence the strength and direction of self-other comparisons (Corcoran et al. 2011). Whether people tend to assimilate to or contrast away from a certain standard is for example significantly influenced by characteristics of the standard, such as its extremity (Herr 1986; Mussweiler et al. 2004a), the personal relationship of self and standard (e.g. Lockwood and Kunda 1997); as well as the cognitive mechanisms that are engaged in the comparison (Mussweiler 2001). Social comparison may result in assimilation if the standard is moderately different or belongs to the same category as the self (Mussweiler et al. 2004a; Mussweiler and Bodenhausen 2002; for reviews see Corcoran et al. 2011; Mussweiler 2003) or lead to contrasting away if the standard is extreme or belongs to an out-group.

Psychological research to date has shed light on many core facets of social comparison processes. Yet, one fundamental question remains unexplored: What are the evolutionary origins of this important facet of human cognition and behaviour? Are we dealing with a uniquely human characteristic that is tightly linked to our self-consciousness, or are social comparisons—or at least rudimentary forms thereof—shared with other species that have evolved complex social relationships? Behavioural observations indicate that animals compare themselves to others to a certain extent. For example, the ability to compare an opponent’s fighting ability to one’s own resource-holding potential is advantageous as harmful fights can be avoided (Searcy and Nowicki 2005). Male chacma baboons (*Papio ursinus*), for instance, utter series of so-called wahoo calls when displaying. The acoustic features of these calls are related to fighting ability (Fischer et al. 2004). Importantly, when two males display at the same time to demonstrate and assess their fighting ability, displays tend to escalate when males appear to have a similar quality, whereas the “weaker” male tends to withdraw when confronted with a male with clearly higher resource-holding potential (Kitchen et al. 2003). Likewise, playback experiments revealed that chimpanzees and lions are able to judge the number of opponents and adjust their responses, i.e. attack or retreat, according to the relative difference of their own and the opponents’ group size (McComb et al. 1994; Wilson et al. 2001).

Further evidence for the assumption that animals are sensitive to relative differences between themselves and others comes from experimental studies. In the so-called “inequity aversion paradigm” (Brosnan and de Waal 2003), subjects refuse to participate in experiments if a partner animal is receiving better rewards for the same task. In these experiments, two animals are seated next to each other and have to complete a certain task, typically exchanging some tokens with a human experimenter, to receive a food reward. If both animals receive the same type of food or if only one animal is present, these exchange tasks are completed reliably. If the partner receives a “better” food reward for doing the same task, however, subjects frequently stop participating and may even show signs of distress and aggression. They seem to be upset by the “unfair” treatment (see Price and Brosnan 2012, for a review). In fact, such sensitivity to inequity in reward distribution has not only been demonstrated for primates, but also for domestic dogs (*Canis familiaris*, Range et al. 2009), as well as carrion crows and ravens (*Corvus corone, C. corax*, Wascher and Bugnyar 2013; but see McAuliffe et al. 2015).

Although previous studies indicate that animals are sensitive to differences in reward outcomes and fighting ability, the psychological mechanisms supporting social comparison processes in animals remain largely unknown. We combined experimental paradigms from social psychology with animal behaviour research methods to explore these processes in more detail. Specifically, we applied insights from human social psychological research on social comparisons to animals, using the co-acting paradigm developed by Seta (1982). In the co-acting paradigm, two human individuals worked independently on the same task, whereby correct responses were accompanied by a feedback sound audible for both individuals. The acoustic feedback significantly influenced subjects’ performances, in such a way that working simultaneously with a slightly superior co-actor led to better performances than working alone or with an extremely better co-actor. Unlike studies that target the effects of comparison processes on self-evaluation, this paradigm assesses the effects of social comparison processes on performance, and thus lends itself for comparative research.

We tested long-tailed macaques in the co-acting paradigm, to explore whether general social comparison processes are apparent in non-human subjects, and to examine whether similar factors influenced the strength and direction of the comparison. Recent studies indicate that macaques appear to be particularly skilled at social tasks and are both pro-social (Massen et al. 2010) and sensitive to inequity (Massen et al. 2012; Hopper et al. 2013). In the current study, the monkeys solved discrimination tasks on a touch screen, while receiving auditory feedback about the performance of a co-actor. We hypothesized that social comparisons are a shared cognitive mechanism that evolved in response to life in a complex society and predicted that the long-tailed macaque subjects respond similarly to variation in the performance of the comparison standard, in this case the co-actor, as humans. We therefore investigated whether social comparisons in monkeys were influenced in similar ways by (a) the relative difference between the subject’s (i.e. the target), and the co-actor’s (i.e. the standard) performance and (b) the strength of the social bond between subject and co-actor. Social comparison consequences in humans critically depend on the similarity of target (i.e. the subject and his/her ability or characteristic) and standard (i.e. the subject to be compared with and his/her ability or characteristic) (Mussweiler 2003). The more similar a human target is to the comparison standard, the more likely this target will assimilate his or her performance, self-evaluations, and affective reactions to the standard. The more dissimilar a human target is to the standard, the more likely he or she will contrast away from the standard. This basic pattern holds for similarity on the performance dimension itself, as well as for similarity on performance-unrelated dimensions such as social closeness. As a consequence, humans tend to assimilate to moderate comparison standards (i.e. standards that are similar to them) on the performance dimensions and contrast away from extreme standards (Mussweiler et al. 2004a). In much the same way, humans tend to assimilate to standards with whom they have a close social bond and contrast away from those with whom they do not have close bonds (Brown et al. 1992; Mussweiler and Bodenhausen 2002).

To test these assumptions in monkeys, we manipulated the co-actor’s alleged performance to be either extremely or moderately different from the actor’s baseline performance ability. Thereby we used upward and downward comparison standards, i.e. the alleged co-actor performed either better or worse than the target subject. Furthermore, we tested subjects with co-actors with whom they either had close (hereafter “affiliates”) or weaker social relationships (“non-affiliates”). This allowed us to test the effects of upward and downward comparison standard and bond strength, and the interaction of the two factors. According to Mussweiler et al. (2004a), subjects should assimilate to a moderate standard and contrast away from an extreme standard; furthermore, they should assimilate to socially close others and contrast away from socially distant others (Brown et al. 1992; Mussweiler and Bodenhausen 2002), resulting in a significant interaction between direction and extremity as well as direction and relationship category.

## Materials and methods

### Ethical statement

All testing was non-invasive, and subjects participated voluntarily. They were not food deprived for testing, and water was always available ad libitum. The monkeys were fed regular monkey chow, fruits, and vegetables twice a day. Their enclosure was equipped with wooden platforms, fire hoses, and several enrichment objects, which were changed on a regular basis. All experiments were performed under the control of experienced veterinarians to ensure that the studies were in accordance with the NRC Guide for the Care and Use of Laboratory Animals and the European Directive 2010/63/EU on the protection of animals used for scientific purposes. In accordance with the German Animal Welfare Act, the study was approved by the Animal Welfare Officer of the German Primate Center (Permit Number 33.9-42502).

### Subjects

Nine long-tailed macaques—three females and six males, aged one to 7 years (Table 1)—participated as test subjects in the experiments. Four additional female monkeys (Lucy, Maja, Selina, Sunny, aged 3–12 years) served as “co-actors” during the test phase of the experiment, but did not perform the discrimination tasks themselves. The monkeys lived in a social group of 35 individuals. They were housed at the German Primate Center in Göttingen and had access to indoor (49 m^2^) and outdoor areas (141 m^2^), which were equipped with trunks, ropes, branches, and other enriching objects.

**Table 1:** Information on test subjects

Name	Sex	Date of birth	Passed	Test participation
Ilias	m	29.12.2012	Yes	Test subject and co-actor
Isaak	m	10.04.2011	Yes	Test subject and co-actor
Lenny	m	10.04.2009	Yes	Test subject and co-actor
Linda	f	22.04.2009	Yes	Test subject and co-actor
Linus	m	16.01.2013	Yes	Test subject and co-actor
Mila	f	07.04.2012	Yes	Test subject and co-actor
Popey	m	08.06.2007	Yes	Test subject and co-actor
Max	m	01.02.2013	Yes	Test subject and co-actor
Sophie	f	03.04.2009	Yes	Test subject and co-actor
Lucy	f	24.02.2011	No	Co-actor
Maja	f	17.10.2007	No	Co-actor
Selina	f	20.05.2008	No	Co-actor
Sunny	f	09.08.2002	No	Co-actor

Name, sex, date of birth, whether they passed the training phases, and whether they participated as test subjects and co-actor, or only as co-actor

For the experiments, the monkeys were lured into a separate cage (2.60 m × 2.25 m × 1.25 m; height × width × depth) adjacent to the indoor enclosure, which could be subdivided into six experimental compartments. Test participation was voluntary, i.e. dependent on the monkeys’ willingness to enter the testing compartment. During the experimental sessions, monkeys received flavoured pellets (touch screen reward: 45 mg sucrose tablet, Sandown Scientific; one per correct answer) and various types of fruits, peanuts, or raisins. The tested monkeys were already experienced in participating in behavioural experiments (e.g. Schmitt et al. 2012; Schloegl et al. 2013) and were trained to use a touch screen computer. Tests were conducted once or twice a day between January and November 2014.

### Experimental setup

**Fig. 1:**
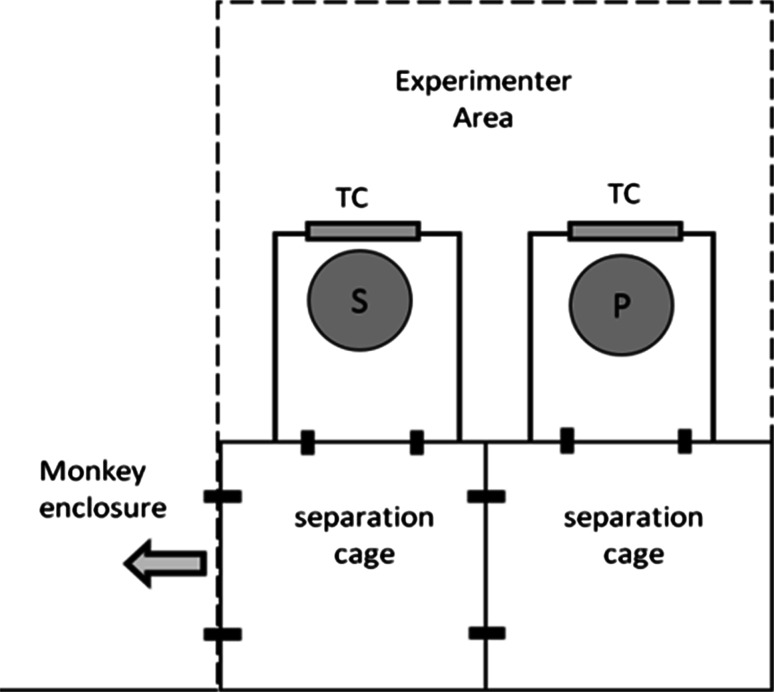
Experimental setup used to test the monkeys. Two touch screen boxes placed next to each other were attached to the separation cages. *TC* touch screen computer, *S* subject, *P* partner

The experimental cage was divided into a separate compartment for the subject and the partner. Each compartment was connected to a box (80 cm × 70 cm × 60 cm; height × width × depth; see Fig. 1) equipped with a touch screen computer (17″ Elo Touchsystem MPRII). The two testing compartments were visually, but not acoustically separated. The subject’s as well as the co-actor’s behaviour was recorded by two cameras on top of each box.

### General procedure

The experimental procedure consisted of three consecutive parts: the training, the pre-test, and the test phase. In all parts, subjects solved discrimination tasks on a touch screen computer. During the training, subjects were habituated to the experimental setup and learned the required two-choice discrimination task. Subsequently, the pre-test was conducted, in which subjects had to transfer the learned discrimination to novel stimuli. The individual results of the pre-test were used to calculate the average performance of each monkey in order to prepare individual feedback playbacks for the test (see paragraph on *Experimental Conditions* for details). In the test phase, subjects solved the same discrimination task as in the pre-test, but with varying feedbacks and varying co-actors.

### Training

**Fig. 2:**
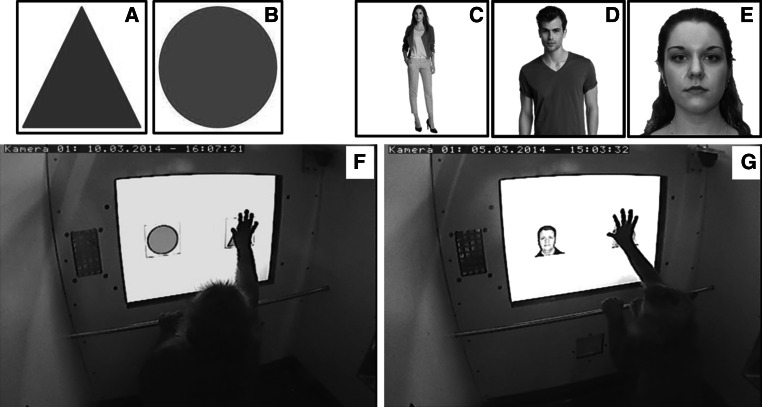
Examples of stimuli used in the discrimination tasks and experimental setup. In the first training phase, the monkeys learned to discriminate between images of triangles (**a**) and circles (**b**) in six different colours on the touch screen (**f**). In the pre-test and test phase, pictures of male and female humans had to be discriminated. In each session, whole-body pictures (**c**), images of the upper half of the body (**d**), and face-only pictures (**e**) were presented on the touch screen (**g**)

Prior to the actual testing procedure, two phases of training (circle/triangle training and male/female training; see Fig. 2) were conducted. To accomplish the training sessions, the subject was separated in one of the two touch screen boxes (itself chose which one it wanted to enter) and performed the task on its own, with no other monkey being in the touch screen box next to it. In these training sessions, subjects solved discrimination tasks in a two-choice paradigm and learned that touching the positive (i.e. correct) stimulus twice resulted in a short “beep” sound (frequency = 300 Hz, duration = 100 ms) and an automatic release of a food pellet (which stimulus served as positive or negative was randomized between subjects). Choosing the negative (i.e. incorrect) image produced an error sound (frequency = 150 Hz, duration = 300 ms), no food reward, and the presentation of a red screen lasting for 1 s. Subsequent to this positive or negative feedback, an inter-trial interval of 2 s followed before the next image-pair appeared. If the monkeys touched the screen during the inter-trial interval, the interval was prolonged by an additional 2 s to ensure that the animals viewed both of the following pictures before touching one of them. If the negative image had been chosen, the same picture pair was presented again as correction trial, until the correct image was chosen. All sessions were created in E-Prime (E-Studio; Version 2.0 Professional) and contained 20 trials and thus, 20 pairs of images, each. The pairing of the stimuli was fixed in advance by utilization of the freely available software “randomizer” (Urbaniak and Plous 2013). The order of appearance of the determined stimulus pairs in each session was randomized automatically by E-Prime. If a monkey left the setup before discriminating at least 14 pairs of pictures, the session was not counted; instead, it was repeated the following day. The monkeys successively had to pass the criterion in one phase before reaching the next phase (see the following description of discrimination training).

### Circle/triangle discrimination

In each trial of the circle/triangle discrimination task, one circle and one triangle were displayed in the centre of the touch screen on a white background with a distance of 11 cm between them (see Fig. 2). As stimuli, images of circles and triangles in six different colours (RGB values: blue 0-128-255, yellow 255-242-0, green 34-177-76, orange 255-127-39, red 255-0-0, and violet 128-0-255) were generated and surrounded by a 269 × 269 pixel-sized black frame using Microsoft Paint (see Fig. 2). Several studies showed that colour vision in Old World monkeys is typically trichromatic with similar peaks of spectral sensitivity as in humans (see e.g. Bowmaker et al. 1991). Thus, it can be assumed that colour vision in long-tailed macaques is comparable to human colour vision and that all six colours were perceived evenly well. By random assignment, the “circle” was the positive category for half of the subjects, for the other half “triangle” was the positive category. The usage of different colours should ensure that subjects learned the category “shape” and not just learned to choose an object of a certain colour. Individuals were trained until they reached a criterion of 75 % correct first choices in three out of six consecutive sessions. As a next step, they were trained in the male/female discrimination-paradigm.

### Male/female discrimination

Subjects were trained in a two-choice paradigm similar to the circle/triangle discrimination but with pictures of (Caucasian) male and female humans as new categories. In each trial, one image of a man and one image of a woman were displayed in the centre of the touch screen on a white background with a distance of 11 cm between them (Fig. 2). As stimuli, pictures of whole persons, pictures of the upper half of the body, and pictures of human faces were used (Fig. 2). These three different types of pictures were applied to increase the difficulty of the task. Images were purchased from an online database and other Internet sources (detailed list of sources see Electronic Supplemental Material). Pictures were chosen to be of preferably similar brightness and colour composition and were as neutral as possible. For example, pictures with plainly visible accessories or men with large beards were excluded, and facial expressions of people on pictures used were neutral. As in the training phase, all stimuli were surrounded by 269 × 269 pixel-sized black frames.

For four subjects, “male” pictures were the positive (rewarded) category; for the other five, female pictures constituted the positive category. Each session contained 20 pairs of images out of a set of 80 (40 male and 40 female). Therefore, for every session, six pairs of whole-body pictures, six pairs of half-body pictures, and eight pairs of face pictures were used. Each picture was shown only once within one session, but in the course of different sessions, each image appeared repeatedly as part of variable pairings. Subjects were trained until they reached a criterion of 70 % correct first choices in three out of six consecutive sessions. This relatively weak criterion should ensure that it was possible for the subjects’ performance to either improve or worsen during the subsequent tests. After reaching the criterion, subjects proceeded to the pre-test sessions. Correction trials were administered as described above. The criterion of the male/female discrimination was reached between the 11th and 27th session (mean = 17.1 sessions).

### Pre-test

In order to prepare individual feedbacks for each subject in the subsequent test sessions, three pre-test sessions were conducted. These were equivalent to the male/female discrimination training described above, but consisted of 10 familiar and 10 novel pairs of images that appeared in random order. Again, subjects performed on their own, without a co-actor in the touch screen box next to them. In addition to the subject’s accuracy, the reaction time (RT), i.e. the time between the appearance of the stimuli and the monkey making a choice, was automatically logged with an accuracy of 0.001 s. This information was used to calibrate the individual playbacks according to the individual performance of the subjects. Two subjects (Ilias and Lenny) performed so well in the pre-tests that there was barely any improvement possible for the subsequent test. For these two monkeys, the three pre-test sessions were repeated with stimuli of 50 % increased brightness, so that they were more difficult to be recognized correctly. Afterwards and based on the new pre-test results, new playbacks were created. After the three pre-test sessions were accomplished, subjects proceeded to the test.

### Experimental conditions

The test procedure was similar to the male/female discrimination pre-test, but using varying feedback conditions and varying co-actors. Each subject was tested in five different conditions: four playback conditions (feedback playbacks of the co-actor apparently performing *moderately better/worse or extremely better/worse*) and a *social control* condition, in which the subjects did not receive any acoustic information about the performance of the co-actor, who was either an affiliate or a non-affiliate. On average, the number of positive feedback tones was 19.4 in the extremely better condition, 5.8 in the extremely worse condition, 16 in the moderately better, and 12 in the moderately worse condition. The playbacks were generated with Audacity (Version 2.0.5) using the positive “beep” sound (frequency = 300 Hz, duration = 100 ms) followed by the click sound of the food-dispenser or the error sound (frequency = 150 Hz, duration = 300 ms). The difference in the frequencies is substantially above the “just-noticeable difference” identified for monkeys (e.g. around 30 Hz, Sinnott 1985). Each playback consisted of 20 feedback sounds (corresponding to one session with 20 trials), and the proportion of positive and negative sounds of each individual playback depended on the corresponding individual performance of the given monkey in the pre-test sessions. Four different kinds of playbacks were prepared for each monkey: (1) “moderately better”: mean performance of the subject + 1 SD; (2) “moderately worse”: mean performance—1 SD; (3) “extremely better”: mean performance + 4 SD; (4) “extremely worse”: mean performance—4 SD.

The time interval between two sounds was adjusted so that it was similar to the inter-trial interval each monkey had produced in the pre-test sessions of the discrimination task and ranged between 3.6 and 6.9 s. Likewise, the maximum and minimum amount of positive and negative sounds that followed each other in the playback was established based on the maximum and minimum amount of correct and incorrect choices made by the given monkey in pre-test sessions. For each of the four types of playbacks, six different versions with varying order of sounds were generated, so that each monkey was able to listen to the same playback only once during the whole testing phase. In total, 24 playbacks were prepared for each subject. As one of the monkeys regularly touched the screen forcefully, the sound produced by that was recorded with a Marantz Solid State Recorder PMD661, and added prior to each feedback sound in playbacks for those subjects who had this specific monkey as co-actor. The five conditions were presented in two blocks of ten sessions each (20 sessions in total per individual) with pseudo-randomized and counter-balanced order of the sessions within a block, i.e. every condition was presented once within each block.

### Co-actors

Each subject was tested with affiliates and non-affiliates as co-actors in two blocks. To control for order effects, half of the animals were tested with affiliates in the first block and the other half was tested with non-affiliates in the first block. The classification into “affiliates” and “non-affiliates” was based on an observation study conducted just before the training started. Based on 435 focal observations collected from 29 individuals that lasted 20 min. each, we calculated the dyadic composite sociality index (CSI; Silk et al. 2006, 2013) for all dyads. This method is frequently used in scientific studies on the sociality of Old World monkeys (e.g. Silk et al. 2006) and provides a reliable measure to classify individual bonds. CSI values were derived from grooming, contact sitting (sitting in close proximity with body contact) as well as social playing, because several juveniles and young adults were among the subjects that spent much time playing. By definition, a CSI of 1 represents the average CSI. The CSI of affiliates was *M* = 4.87 (SD = 3.53) and of non-affiliates *M* = 0.36 (SD = 0.34). The number of co-actors used per subject ranged between 1 and 4 per category and was mainly determined by the number of affiliates or non-affiliates an individual had, as well as by their availability, i.e. willingness of those monkeys to enter the test cage.

### Test procedure

For the test sessions, first the focal animal was separated in one of the two touch screen boxes. The focal animal chose which of the two boxes it wanted to enter. Subsequently, one of the corresponding co-actors was led into the other touch screen box. As most of the subjects had more than one potential affiliate or non-affiliate, the one who entered the test cage first was chosen as co-actor for that session. To ensure that the focal animal knew which individual was in the adjacent touch screen box, they were given brief visual access through opening the slider between the compartments a few inches before the actual test sessions started. In some of the sessions, the focal subject and the co-actor entered the testing cages together and were subsequently separated into the two touch screen boxes, i.e. no further visual access was needed. After ensuring that the subject had seen the co-actor and each of them was situated in his/her respective touch screen box, the slider was closed and no more visual access between the animals was possible for the entire session. As soon as subject and co-actor were visually isolated from each other, the co-actor was fed with raisins to stay calmly in the test compartment, and the playback of the appropriate condition was started using a Marantz Solid State Recorder PMD661. This was connected to a MIMI loudspeaker that was attached to the touch screen box of the co-actor, so that the focal subject was led to believe the co-actor would be performing the discrimination task. To ensure that the test subjects could perceive the composition of the co-actor’s performance, first the feedback of 20 trials was played. During this time, the subject was not allowed to perform at the touch screen. After the first 20 playback sounds, the experimenter switched on the subject’s screen and the same 20-trial feedback was played again. The subject now performed its own discrimination task, while the feedback of the co-actor was played. Again, each session consisted of ten familiar and ten unfamiliar pictures. The number of correct first choices and correctly chosen novel stimuli and errors (including correction trials) was measured, as well as the reaction time (RT) between the appearance of the stimuli and the monkey making a choice. The performance in the correction trials was not considered, i.e. these trials were excluded from analysis.

### Data analysis

The performance was assessed as the number of “correct” responses in a total of 3585 trials of nine monkeys. In 2867 trials, subjects received feedback about the alleged performance of the co-actor; in the remaining 718 trials, the co-actor was also present but no feedback was provided (“social control” condition). We included direction (better, worse), extremity (moderate, extreme), and relationship (affiliate, non-affiliate) as fixed factors of interest, including the interaction between direction and extremity, and direction and relationship category. Further, stimulus novelty (novel/familiar) and block (1, 2) were used as fixed control factors, and subject ID as a random factor. We ensured that assumptions were met (see ESM). For the analysis of accuracy, we used generalized linear mixed model analysis (GLMM) with binomial error structure and subject-specific random effects. We also included RT as a potential predictor of accuracy. For the analysis of RT, we used quantile regression for longitudinal data (Koenker 2004, see ESM), as the distribution of RT was extremely right skewed. The analysis of RT is based on 3584 trials, as E-Prime did not stamp the time for one trial, yielding 2866 trials in the feedback and 718 in the social control condition. Finally, we compared the performance in the social control condition to that in the all of the feedback conditions combined.

## Results

### Influence of condition and relationship

**Fig. 3:**
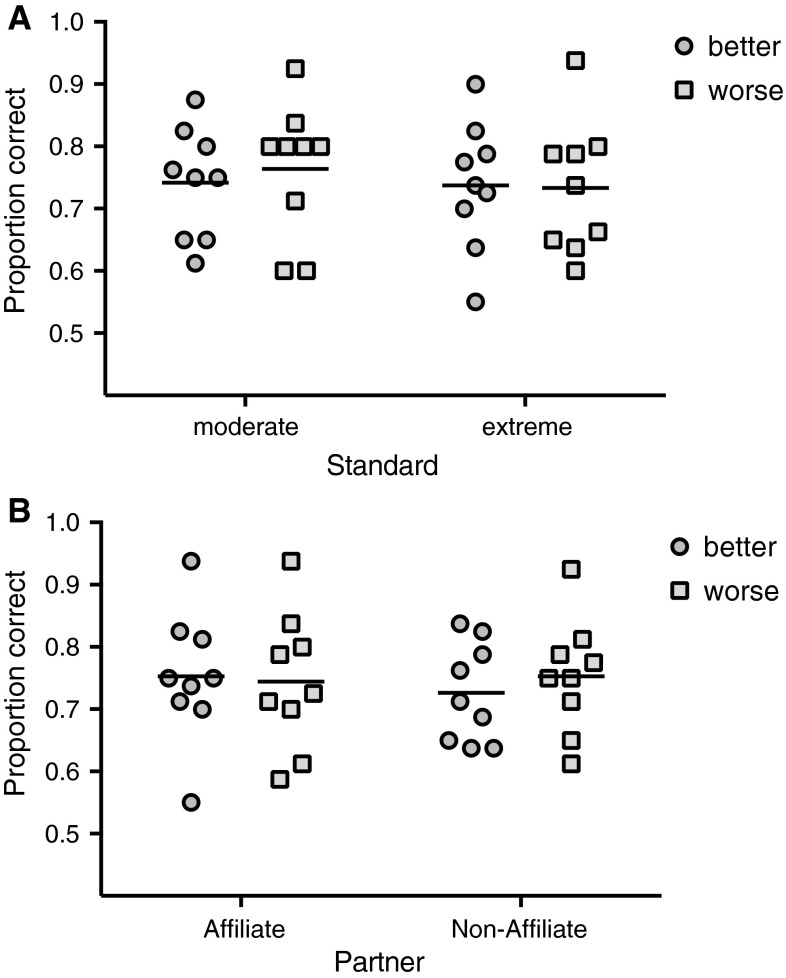
**a** Effect of standard extremity and direction of comparison. Individual mean proportions of correct responses in relation to extremity (moderate vs. extreme) and direction (*circle*: better; *triangle*: worse). *Bars* indicate overall means per condition. **b** Effect of relationship and direction of comparison. Individual mean proportions of correct responses in relation to relationship (affiliate vs. non-affiliate) and direction (*circle*: better; *triangle*: worse) are given. *Bars* indicate overall means per condition

**Fig. 4:**
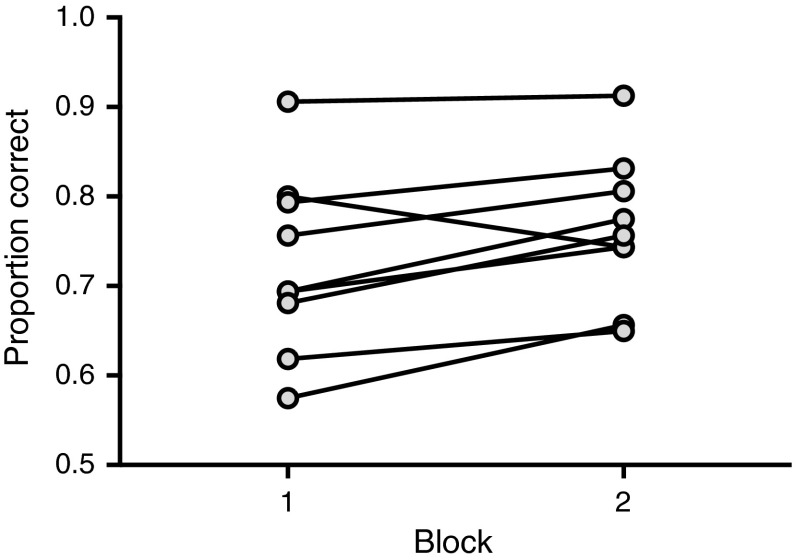
Differences in performance in the two experimental blocks. Individual mean proportion correct responses in the first block (B1) and second block (B2) are given. *Bars* indicate overall means per condition

Neither of the two interactions nor the main factors of interest affected the subjects’ accuracy in the tests (Table 2, Fig. 3a, b). In contrast, subjects did better in the second compared to the first block of the study (Block 1: *M* = 72.4 % vs. Block 2: 76.4 %, *p* = 0.007). More specifically, eight of the nine subjects showed a better performance in the second compared to the first block (Fig. 4). Interestingly, the level of familiarity of the pictures (novel vs. familiar pictures) had no apparent influence on the performance of the monkeys (novel *M* = 74.2 % vs. familiar 74.7 %, *p* = 0.813). Reaction time did not predict accuracy levels (Table 2).

**Table 2:** Effects of the different predictor variables on accuracy

Coefficients	Estimate	SE	*z* value	*p*(>|*z*|)
(Intercept)	0.9081	0.2117	4.289	1.8e−05***
Reaction time	0.0084	0.0058	1.442	0.149
Relationship (affiliate)	0.1292	0.1229	1.052	0.293
Stimulus novelty (familiar)	0.0207	0.0874	0.237	0.813
Block (2nd block)	0.2355	0.0877	2.685	0.007**
Direction (worse)	0.0622	0.1504	0.413	0.679
Extremity (moderate)	0.0166	0.1577	0.105	0.916
Direction (worse) × relationship (affiliate)	−0.1871	0.1750	−1.069	0.285
Direction (worse) × extremity (moderate)	0.1428	0.1748	0.817	0.414

Estimates for the predictor variables with reference category, standard errors, *z* values, and *p* values obtained from the GLMM analysis. *N* = 2866 trials with nine subjects

*** *p* < 0.001; ** *p* < 0.01

**Fig. 5:**
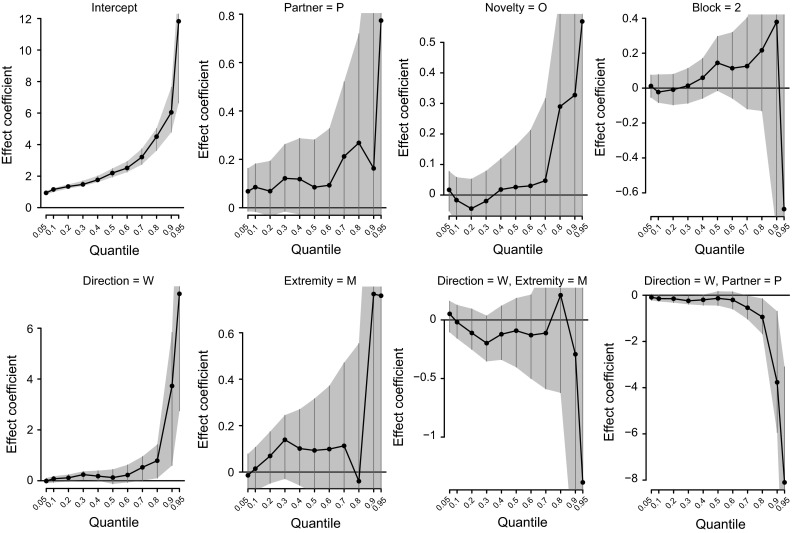
Effect of the key predictor variables on the location of the quantiles. A positive shift in the location of the upper quantiles indicates that long RTs occur more frequently. *Shaded areas* indicate 95 % confidence intervals; nonzero effects can be inferred when confidence intervals do not overlap with the null value

For the reaction time, we found evidence for an interaction between relationship quality and direction of the standard on the occurrence of long RTs. Specifically, long reaction times occurred more frequently when a non-affiliate performed worse, compared to when he performed better than the subject. When subjects were tested with an affiliate, there were no discernible effects of partner performance on reaction time (Fig. 5). The occurrence of long reaction times affected the location of the upper quantiles. For instance, the 80 % quantile was 5.1 when a non-affiliate performed worse, and 4.3 s when he performed better. For affiliate co-actors, there was no marked effect on the occurrence of long latencies (worse: 4.4, better: 4.6 s). Bayesian quantile regression reaffirmed our findings on the upper quantiles of reaction time (see ESM).

**Fig. 6:**
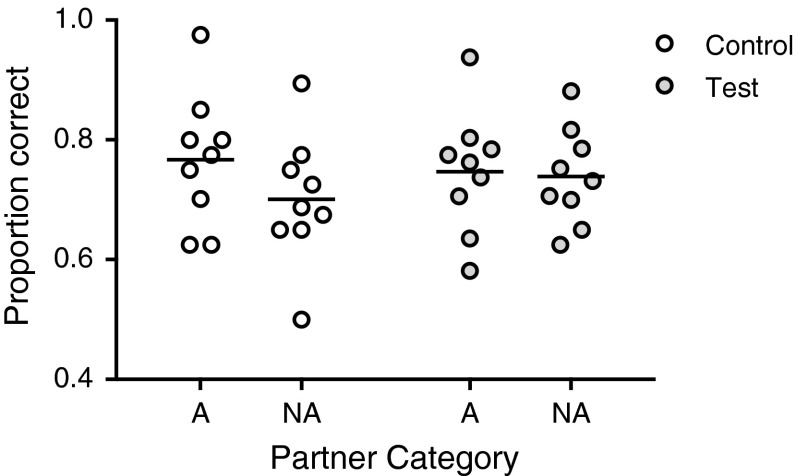
Comparison of social control and feedback conditions. Individual mean proportion correct responses in the social control (*open circles*) and the experimental feedback conditions (*light grey circles*) separately for sessions with an affiliate (A) and a non-affiliate (NA) present as co-actor. *Bars* indicate overall means per condition

We next investigated whether the accuracy in the experimental conditions, i.e. when feedback was provided, differed from that in the social control condition, where an affiliate or non-affiliate was present, but no feedback was provided. We found no main effect of feedback, a significant effect of relationship quality, and no interaction between feedback and relationship (Table 3). An inspection of the data suggested that the effect of relationship quality only became apparent in the control condition, and the test lacked the power to detect the interaction. Stratified models revealed evidence that subjects performed better in the social control condition when an affiliate was present compared to when a non-affiliate was present (*M* = 77.8 % correct vs. 71.1 % correct, *p* = 0.042), while there was no evidence for a difference in the feedback conditions (*M* = 75.8 vs. 75.0 % correct, *p* = 0.627; Fig. 6). There was also no evidence for effects of the predictors under investigation on RT (see supplementary information).

**Table 3:** Effect of the social control condition and relationship quality on performance

Coefficients	Estimate	SE	*z* value	*p*(>|*z*|)
(Intercept)	0.9024	0.2064	4.373	1.23e−05***
Relationship (affiliate)	0.3535	0.1730	2.044	0.041*
Social control (feedback)	0.1999	0.1328	1.506	0.132
Social control (feedback) × relationship (affiliate)	−0.3111	0.1936	−1.607	0.108

Estimates for the predictor variables (with reference category), standard errors, *z* values, and *p* values obtained from the GLMM analysis

*** *p* < 0.001; * *p* < 0.05

## Discussion

The performance of the long-tailed macaques did not conform to the predicted pattern, in the sense that in terms of accuracy, they would have assimilated to moderate standards or affiliates, while contrasting away from extreme standards and non-affiliates. This result cannot be explained by an apparent lack of sensitivity to relationship quality or partner performance, as the reaction time was affected by both factors. Specifically, when tested with a non-affiliate, subjects more frequently showed long RTs when the co-actor was a non-affiliate who was doing worse, but responded within the normal range when he was doing better than the subject. When tested with an affiliate partner, performance did not vary in relation to partner performance. This may indicate that social comparison processes do occur in the presence of perceived competitors and that these comparison processes lead to assimilation, at least in terms of the rate at which monkeys perform the experiments. Alternatively, subjects found the presence of the non-affiliate disruptive, yet were piqued by the non-affiliate’s apparent better performance, so that they kept responding at a fast rate. This assumption also fits with the observation that their performance in the social control condition was slightly worse when the co-actor was a non-affiliate. Yet, reaction times in the social control did not vary in relation to relationship quality, indicating a rather intricate interaction between the occurrence and type of feedback as well as the relationship with the co-actor.

The extremity of the standard affected neither accuracy levels nor reaction time, raising the question whether the difference in the number of positive feedback tones provided in the moderate and extreme standards was perceptible. On average, this difference was 3.4 tones/session in the better direction and 6.2 tones/session in the worse direction. Since the difference in the number of positive feedback tones compared to the “self” (on average) was only 2 tones/session, this explanation seems unlikely. Instead, the results suggest that the direction of the difference is more salient than the magnitude of the difference. Furthermore, subjects performed significantly better in the second compared to the first block of the experiments, indicating that they became more skilled at the task.

It is important to note that the original study by Seta (1982) did not involve the distribution of reward, which may also have affected the outcome in our study. Perhaps, for the monkeys, the positive feedback signalled the distribution of a reward, while for the humans, the positive feedback was more indicative of the success of the co-actor. Therefore, this (necessary) variation in experimental design may have effectively caused a shift in attention, which may have affected the behaviour in the subjects. It would be interesting to study the effect of immediate reward compared to indirect information about performance in follow-up studies in humans.

Because rank plays a significant role in this rather despotic species (Thierry 2007), it would have been desirable to include rank difference in the analyses. Yet, because rank was not a fixed factor of interest, the data set was not balanced and only qualitative assessments are possible for those five subjects that were tested with both higher- and lower-ranking co-actors. An inspection of the 70, 80, and 90 % quantiles revealed no consistent pattern (data not shown). The same holds for relatedness between the subjects of the different test pairs. The animals live in a large social group, which has been housed at the German Primate Center for over 30 years, including many related individuals. Thus, some of the test pairs were closely related (e.g. siblings), more distantly related (cousins), or not related at all. However, each subject was tested with several co-actors of varying relatedness, as we had categorized them as affiliates or non-affiliates based on behavioural observations. These behavioural observations had shown that the subjects had strong or weak social relationships with the other group members irrespective of their relatedness, e.g. with subjects of the same sex. Focusing on affiliate/non-affiliate partners, we did not have a balanced distribution of related and unrelated test pairs to do meaningful statistical analysis.

Although we found evidence that the monkeys were sensitive to partner identity and performance, the results did not conform to the predicted pattern. Rather than sharing the specific social comparison processes resulting in assimilation and contrast effects with humans, other mechanisms might be at work in non-human primates (or at least long-tailed macaques). Specifically, both competitive drive and social facilitation may have affected subjects’ behaviour in the experiments. Social facilitation refers to the finding that the mere presence of another individual may enhance (Addessi and Visalberghi 2001; Galloway et al. 2005) or inhibit the motivational state of a subject (Zajonc 1965). Social facilitation can also affect task performances, and this effect is known to play a role in humans (e.g. Travis 1925) as well as in other species, such as capuchin monkeys (Dindo et al. 2009), rats (*Rattus norvegicus*; Levine and Zentall 1974) or cockroaches (*Blatta orientalis*; Zajonc et al. 1969). In which way the presence of a partner affects performance is assumed to depend on the relationship to the other (De Castro 1994). Huguet et al. (2014) found that social rank, age, and sex of the surrounding individuals influenced the reaction time of subjects doing a computerized task. Thus, in our study, the presence of a non-affiliate may have been perceived as disruptive, but competitive drive may have kept subjects focused on the task when the co-actor was performing better than them. This is in line with a recent experimental study by Engelmann et al. (2015), who found that chimpanzees retrieved more food items from an apparatus in a co-action condition where another individual was working on an identical apparatus next to the subject, compared to a mere presence condition in which another individual merely watched the subject retrieving food. This result pattern indicates that the competitive context induced concern for the performance of the co-actor and increased subjects’ own performance motivation.

Future studies should explore the putative effects of competition and social facilitation (or rather social disruption) in more detail, and the co-acting paradigm appears to be a promising research avenue (see Martin et al. 2014) in this regard. Ultimately, larger sample sizes will be needed to better understand the mechanisms underpinning social comparison processes in non-human animals. Because single institutions rarely house sufficient numbers of subjects, future research should increasingly be built on coordinated efforts by larger consortia (MacLean et al. 2012).

As an interesting side aspect, this study shows that Old World monkeys are able to categorize pictures of human males and females and generalize this knowledge to novel and unfamiliar pictures. Troje et al. (1999) demonstrated that pigeons (*Columba livia*) are capable of discriminating human male and female faces. Moreover, pigeons can distinguish familiar and unfamiliar humans by interpretation of facial features (Stephan et al. 2012). In primates, several studies showed that Old World monkeys and apes, as well as one species of New World monkeys (capuchin monkeys) are able to discriminate faces of either conspecifics (Boysen and Berntson 1989; De Waal et al. 2000; Pokorny and de Waal 2009) or humans (Boysen and Berntson 1986; Keating and Keating 1993; Martin-Malivel and Fagot 2001; Martin-Malivel and Okada 2007). Furthermore, Paukner et al. (2010) showed that infant rhesus macaques have a preference for pictures of female human faces, suggesting that they were able to differentiate between human sexes. That the long-tailed macaques in the present study were capable of categorizing male and female humans supports the findings of Little et al. (2008) who demonstrated that human faces share many of the sexually dimorphic characteristics that are displayed by macaque faces.

In sum, we believe that it is worthwhile to explore the mechanisms supporting social comparison processes further, as there is evidence from a variety of taxa that animals are able to compare themselves to others to a certain degree. For instance, male guppies (*Poecilia reticulate)* choose to solicit females surrounded by males that were less colourful than they were themselves (Gasparini et al. 2013). Furthermore, the strength of this preference was negatively correlated with the male’s own level of ornamentation. In convict cichlids (*Amatitlania nigrofasciata)* females prefer males that are one third larger than they are themselves (Dechaume-Moncharmont et al. 2013). Thus, from cockroaches over fish to non-human primates, subjects are influenced by comparisons to others at least at a basic level. Nevertheless, the elaborate social comparison processes found in humans may be a derived feature of our own species.

## Electronic supplementary material

Supplementary material 1 (PDF 422 kb)
