# Supplementary material for: Do monkeys compare themselves to others?
Source: Anim Cogn. 2015 Nov 28;19:417–28. doi: 10.1007/s10071-015-0943-4 (PMC4751161; doi:10.1007/s10071-015-0943-4)
Supplement: Supplementary file 1 — Supplementary material 1 (PDF 422 kb) [file 10071_2015_943_MOESM1_ESM.pdf]

Do monkeys compare themselves to others?

Electronic Supplement

Vanessa Schmitt Ira Federspiel Johanna Eckert Stefanie Keupp
Laura Tschernek Lauriane Faraut Richard Schuster Corinna Michels
Holger Sennhenn-Reulen Thomas Bugnyar Thomas Mussweiler
Julia Fischer

Corresponding Author: Julia Fischer,
Cognitive Ethology Laboratory,
German Primate Center, Göttingen,
Email: jfischer@dpz.eu

Contents

1 Sources of Stimuli for the Male/Female Discrimination Task **2**

2 Modeling Repeated Binary Outcomes **3**

3 Modeling Quantiles of Repeated Reaction-Times **4**
3.1 Basics 4
3.1.1 Quantile Regression 5
3.2 Modeling Quantiles of Repeated Reaction-Times 6
3.2.1 Selection of Penalty Parameter 7
3.2.2 Bootstrap 95% Confidence-Intervals with Dependence Re-sampling 7
3.2.3 R-Functions 9
3.2.4 Detailed Results 12
3.3 Verification Using Bayesian Quantile Regression of Repeated Reaction-Times 35

References **36**

1 Sources of Stimuli for the Male/Female Discrimination Task

Stimulus category	Type of source	Link
Faces	Park aging mind laboratory (online database)	http://agingmind.utdallas.edu/stimuli
Half bodies	Various internet sources	http://www.witt-weiden.de<br>http://www.baur.de<br>http://www.sheego.de<br>http://www.hm.com<br>http://www.pullandbear.com<br>http://www.urbanoutfitters.de<br>http://www.albamoda.de<br>http://store.americanapparel.net<br>http://www.bandyshirt.com<br>http://www.peterhahn.de<br>http://www.easy+D66youngfashion.de<br>http://www.sachenonlinekaufen.de<br>http://content.yancor.de<br>http://www.arqueonautas.de<br>http://www.trachten24.eu<br>http://www.kademo.de
Whole bodies	Various internet sources	http://store-de.hugoboss.com<br>http://www.hm.com<br>http://www.esprit.de<br>http://www.c-and-a.com<br>http://www.kleider-kunst.de<br>http://www.soliver.de<br>http://www.michaelax.de<br>http://www.burdastyle.de<br>http://www.wellner.modehaus.de

Table S1: Sources of stimuli for the male/female discrimination task.

2 Modeling Repeated Binary Outcomes

Success outcomes were analyzed using mixed-effects models for binary response variables with Logit link-function.

In a first step, Generalized Estimation Equation (GEE) models with first-order auto-correlation structure (AR(1))are applied. Since the absolute auto-correlation parameter $|\rho|$ was only very small (< 0.05), we conclude that there is only negligible dependence between the trials in one session for one subject and consequently use models with an independence correlation-structure, what results in applying ordinary generalized linear mixed models (GLMMs).

For the specification of GLMMs with binomially distributed (binary) outcomes with the Logit link-function, R (R Core Team, 2015) library `lme4` (Bates et al., 2015) was applied, in especially function `glmer()` was used to fit the models.

Selection of random slopes was performed via comparison of variances of random slopes and random intercepts. For the models without *social control*, only the random slope for variable *extremity* provided considerable variance (about 1/3 of the variance explained by the random intercept). Therefore we report results for the model with a random slope for extremity. For the model with social control, none of the explanatory variables yielded a relevant random slope variance, and consequently the random intercept model is reported.

3 Modeling Quantiles of Repeated Reaction-Times

3.1 Basics

The mean is a centrality parameter of a distribution function, as for example parameter μ in the Normal distribution:

$$Y \sim \text{Normal}\left(\mu, \sigma^2\right) \quad \Rightarrow \quad f_{\mu,\sigma}\left(Y = y\right) = \frac{1}{\sqrt{2\pi} \cdot \sigma} \cdot \exp\left(\frac{\left(y-\mu\right)^2}{\sigma^2}\right)$$

Parameter σ is a non-centrality parameter controlling the deviation from the mean. σ is called *standard deviation*, and σ^2 is called *variance*.

The *cumulative density function (cdf)* $F_{\mu,\sigma}\left(Y = y\right)$ takes the value of the integral of the density function $f_{\mu,\sigma}\left(Y = y\right)$ until y:

$$F_{\mu,\sigma}\left(Y = y\right) = \int_{-\infty}^{y} f_{\mu,\sigma}\left(Y = z\right) \, \mathrm{d}z.$$

It's value is a probability $\tau \in [0, 1]$, and it's inverse function $Q_{\mu,\sigma}\left(\tau_Y\right) = F^{-1}_{\mu,\sigma}\left(Y = y\right)$ is called the *quantile function* specifying the τ quantile of Y which will be denoted here by τ_Y. For a given y, the cdf gives the probability of the random variable being equal or smaller than y. For a given probability τ, the quantile function gives the value of the random variable where the cdf has reached this probability. Figure **??** illustrates the relationship between $f_{\mu,\sigma}\left(Y = y\right)$, $F_{\mu,\sigma}\left(Y = y\right)$ and $F^{-1}_{\mu,\sigma}\left(Y = y\right) = Q_{\mu,\sigma}\left(\tau_Y\right)$.

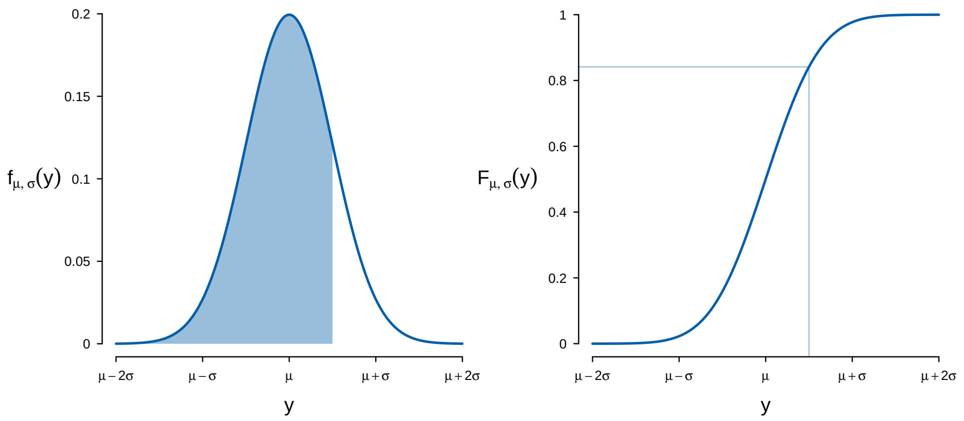

Figure S1: Relationship between $f_{\mu,\sigma}\left(Y = y\right)$, $F_{\mu,\sigma}\left(Y = y\right)$ and $F^{-1}_{\mu,\sigma}\left(Y = y\right) = Q_{\mu,\sigma}\left(\tau_Y\right)$.

In the *linear regression model* based on data $\left(y_i, x_i\right)$ with continuous response variable y and covariate x, we get to the regression relationship:

$$y_i = \mu\left(x_i, \boldsymbol{\beta}\right) + \epsilon_i,$$

where $\mu(x_i, \boldsymbol{\beta}) = E(Y_i|x_i, \boldsymbol{\beta})$ is a regression predictor for the expectation/mean of Y_i, conditional on covariate observation x_i and formed in terms of a linear relationship between x_i and the regression coefficients-vector $\boldsymbol{\beta}$, such as the *univariate linear regression model*:

$$\mu(x_i, \boldsymbol{\beta}) = \beta_0 + x_i\beta_1,$$

with regression coefficients-vector $\boldsymbol{\beta} = (\beta_0, \beta_1)^\top$. For models with more covariates x_l, $l = 1, \ldots, p$, the linear predictor is expanded by the respective model terms $x_l\beta_l$.

3.1.1 Quantile Regression

The idea of *quantile regression* is to let the regression coefficients-vector $\boldsymbol{\beta}$ be potentially different for different quantiles of the response. Instead of specifying only one single model for the mean, quantiles $Q(\tau_Y)$ of Y are specified separately by linear regression predictor functions $q(x, \boldsymbol{\beta}_\tau)$ of x and a τ-specific regression coefficient-vector $\boldsymbol{\beta}_\tau$:

$$Q_{\boldsymbol{\beta}_\tau}(\tau_Y|x_i) = q(x_i, \boldsymbol{\beta}_\tau) + \epsilon_{\tau,i},$$

for $\tau \in (0, 1)$.

We may the again use the univariate linear regression model form to specify the coefficients separately:

$$q(x, \boldsymbol{\beta}_\tau) = \beta_{0,\tau} + x_i\beta_{1,\tau}.$$

For models with more x_l, $l = 1, \ldots, p$, the linear predictor is again expanded by the respective τ-specific model terms $x_l\beta_{l,\tau}$. The linear regression predictor is then conveniently expressed in matrix form by:

$$\mathbf{x}_i^\top \boldsymbol{\beta}_\tau = \beta_{0,\tau} + x_{1,i}\beta_{1,\tau} + x_{2,i}\beta_{2,\tau} + \ldots + x_{p,i}\beta_{p,\tau}.$$

The interpretation of effects on quantile of Y is analogously to the interpretation of effects on the mean of Y. For a binary covariate $x_j \in \{0, 1\}$, the effect $\beta_{j,\tau}$ is the difference between the τth quantiles of Y in the population where $x_j = 0$, and the population where $x_j = 1$.

3.2 Modeling Quantiles of Repeated Reaction-Times

Quantile Regression of repeated reaction-times is the approach that satisfied the assumptions on the statistical model:

1. Linear Mixed Effects Regression (Effects for Direction 0.075, $p = 0.011$, and Direction:Partner -0.082, $p = 0.063$) was not suitable since residuals were homoscedastic, but visual inspection showed them to be not satisfactorily approximately Gaussian distributed.

2. Parametric alternatives by applying Generalized Linear Mixed Effects Models (GLMMs) with log-Gaussian, inverse Gaussian, or Gamma distributed error assumptions showed only minor improvements in terms of the residual distribution.

3. The results from a semi-parametric Cox Proportional-Hazards (Cox PH) model with a random intercept term for Subjects affirmed the results from previous alternative modeling strategies for Direction and Direction:Partner, which indicated a valid mechanism in the data. The proportional hazards assumption was checked using tests for non-zero slope of Schoenfeld residuals vs time in a Cox PH model without random intercept (due to non-available software to do so for the model with random intercept). There was no indication for the violation of the PH assumption. However, when a robust variance estimator was applied, we got strong indications that the effects vary across reaction-time.

4. Parametric survival time analysis techniques, such as the Weibull Accelerated Failure Time-(AFT)-model, showed again no satisfactory distributional fit.

In especially the violation of the proportional hazards assumption led us to explore the complete distribution of the reaction-times.

The applied approach is similar to the strategy introduced in Koenker (2004). We consider the random intercepts-effects model for reaction-times Y:

$$Q_{\boldsymbol{\beta}_\tau}\left(\tau_Y | x_{ij}\right) = \mathbf{x}_{ij}^\top \boldsymbol{\beta}_\tau + \beta_{0,\tau,i} + \epsilon_{\tau,ij},$$

where subscript $i = 1, \ldots, n$ is indexing individual subjects, and subscript $j = 1, \ldots, n_i$ is indexing the n_i distinct measurements made on the ith subject. The overall number of observations is $N = \sum_{i=1}^{n} n_i$.

The subject- and quantile-specific intercepts $\beta_{0,\tau,i}$ "capture some individual specific source of variability, or unobserved heterogeneity, that was not adequately controlled for by other covariates in the model" (Koenker, 2004). The variation in the values for $\beta_{0,\tau,i}$ is known as the random intercept variance from mixed effects regression models.

Using the so-called *check-function*:

$$\rho_\tau\left(u\right) = u \cdot \left(\tau - \mathrm{I}_{\{u<0\}}\right),$$

one has to solve the optimization problem:

$$\hat{\boldsymbol{\beta}}_\tau = \arg\min_{\boldsymbol{\beta}} \left\{ \sum_{i=1}^{n} \sum_{j=1}^{n_i} \left(\rho_\tau \left(y_{ij} - \left(\mathbf{x}_{ij}^\top \boldsymbol{\beta}_\tau + \beta_{0,\tau,i} \right) \right) \right) + \lambda_\tau \sum_{i=1}^{n} ||\beta_{0,\tau,i}||_1 \right\},$$

to yield parameter estimates $\hat{\boldsymbol{\beta}}_\tau$, where $\boldsymbol{\beta}_\tau$ also includes $\beta_{0,\tau,i}$.

For the quantile loss function, it is convenient to consider the L_1 penalty $||\beta_{0,\tau,i}||_1 = |\beta_{0,\tau,i}|$ in place of the conventional L_2 penalty. The random effects estimator is viewed as a penalized weighted absolute residual estimator: “For $\lambda_\tau \to 0$ we obtain the fixed effects estimator [...], while as $\lambda_\tau \to \infty$ the $\hat{\beta}_{0,\tau,i} \to 0$ for all $i = 1, \ldots, n$ and we obtain an estimate of the model purged of the fixed effects” (Koenker, 2004). The shrinkage of the $\hat{\beta}_{0,\tau,i}$ is advantageous in controlling the variability introduced by the large number of estimated $\beta_{0,\tau,i}$ parameters, and the inverse $\frac{1}{\lambda_\tau}$ of the penalty parameter λ_τ is interpreted as being proportional to the random intercept variance.

The above model is implemented in R (R Core Team, 2015) library `quantreg` (Koenker, 2015), in especially by the function `rq.fit.lasso()`.

3.2.1 Selection of Penalty Parameter

We use repeated 10-fold cross-validation to reduce the randomness of the cross-validated selection of λ_τ. For evaluating the predictive performance of the in-bag estimation of the out-of-bag sample, we use the average τ-weighted error (ATWE) (Haupt et al., 2011):

$$\text{ATWE}(\tau) = \frac{1}{n_p} \sum_{i=1}^{n_p} \rho_\tau \left(y_i - \left(\mathbf{x}_{ij}^\top \hat{\boldsymbol{\beta}}_\tau + \hat{\beta}_{0,\tau,i} \right) \right).$$

This grid search for $\hat{\lambda}$ is performed on a proposal vector given by $\lambda = 0.01, 1, 2, \ldots, 30$. Since there is no contextually meaningful standard of comparison for the selection of any subject as the reference subject, and we want to get the direct interpretation of the intercept as the unpenalized population mean, no reference subject is selected, and the deviation from the population mean for each subject is introduced by penalized dummy coefficients as the random component. Without any stabilization introduced by a minimum of penalization, this parametrization of the random component is non-identifiable. So the minimum of the penalty parameter proposal vector is chosen as 0.01. If this minimal proposal value is selected, this will have only a negligible consequence on the estimation of fixed effects in comparison to an unpenalized approach with dummy-coded subjects as fixed effects. As can be seen in Section 3.2.4, this minimum penalty parameter proposal is never selected in our analyses.

3.2.2 Bootstrap 95% Confidence-Intervals with Dependence Re-sampling

For blocks consisting of all observations of one subject i, all paired elements $(y_{ij}, \mathbf{x}_i)$ are re-sampled with replacements, resulting in a bootstrap sample of size n_i for subject i. This is performed for each subject $i = 1, \ldots, n$ and the block results are merged

to one global bootstrap sample containing N observations. The estimated coefficients from this sample are stored, and the procedure is replicated k times. The lower 2.5% and the upper 97.5% empirical quantiles from the k estimates for each coefficient forms the 95%-bootstrap confidence-interval. This strategy is known as the *Percentile Bootstrap*. The combined approach formed by block-dependent re-sampling and Percentile Bootstrap performed best in terms of truth coverage and length of confidence interval in a simulation study by Karlsson (2009), where several strategies to get to confidence intervals for coefficients in quantile regression on longitudinal data were compared.

3.2.3 R-Functions

Following function have been written in order to have a full implementation of the approach as described above.

Check-Function and Average τ-Weighted Error:

Input arguments:

- `y`: vector of response values
- `pre`: linear predictor values as predictor for `y`
- `tau`: quantile of the response variable

Return argument from `check_function()`: numeric vector containing values from the check-function.

```
check_function <- function(pre, y, tau){
  u <- y - pre
  return(as.numeric(u * (tau - as.numeric(u < 0))))}
```

Return argument from `atwe()`: numeric value containing the mean Average τ-Weighted Error.

```
atwe <-function(pre, y, tau){
  res <- check_function(pre = pre, y = y, tau = tau)
  return(mean(res))}
```

Replicated 10-fold cross-validation

Input arguments:

- `k`: number of replications of 10-fold cross-validation
- `tau`: quantile of the response variable
- `lambda`: vector of penalty parameter proposals
- `y`: vector of response values
- `X`: design matrix for fixed effects
- `Z`: design matrix for random effects
- `ID`: subject identifier

Return argument: numeric matrix containing the cross-validated prediction errors in each outer replication loop (rows) and each of the 10 testing samples (columns).

```
kfold10fold <- function(k, tau, lambda, y, X, Z, ID){
  dat <- as.data.frame(cbind(X, Z))
  fmla <- as.formula(paste("y_in ~ -1 +", paste(names(dat), collapse= "+")))
  result <- matrix(nrow = length(lambda), ncol = k*10, 0)
  index_i <- vector("list", length(levels(ID)))
  for(i in 1:length(index_i)){
    index_i[[i]] <- which(ID == levels(ID)[i])}
  count <- 1
  for(j in 1:k){
    index <- rep(0, length(y))
    for(j2 in 1:length(index_i)){
      ho <- index_i[[j2]]
      ho <- sample(ho)
      n_i <- length(ho)
      n_in <- floor(n_i/10)
      index_i_1to10 <- rep(1:10, each = n_in)
      n_missing <- n_i - length(index_i_1to10)
      if(n_missing > 0.5){
        index_i_1to10 <- c(index_i_1to10, 1:n_missing)
      }
      index[ho] <- index_i_1to10
    }
    for(l in 1:10){
      index_in <- which(index == l)
      index_out <- which(index != l)
      dat_in <- dat[index_in, ]
      y_in <- y[index_in]
      dat_out <- dat[index_out, ]
      y_out <- y[index_out]
      for(i in 1:length(la)){
        m <- rq(fmla, lambda = c(rep(0, ncol(X)), rep(lambda[i], ncol(Z))),
                method = "lasso", tau = tau, data = dat_in)
        pre <- predict(m, newdata = dat_out)
        result[i, count] <- atwe(pre = pre, y = y_out, tau = tau)
      }
      count <- count + 1
    }
  }
  return(result)}
```

Bootstrap 95%-Confidence-Interval:

Input arguments:

- `k`: number of bootstrap samples
- `tau`: quantile of the response variable
- `lambda_min`: penalty parameter value where the minimum prediction-error was reached during the cross-validation procedure
- `y`: vector of response values

- `X`: design matrix for fixed effects
- `Z`: design matrix for random effects
- `ID`: subject identifier

Return argument: list of length two containing numeric matrices for the lower (list-element `lower`) and the upper (list-element `upper`) CI-boundaries. Each list-element is a matrix with `k` rows and the number of columns given by the number of fixed coefficients.

```
ci95 <- function(k, tau, lambda_min, y, X, Z, ID){
  fmla <- as.formula(paste("y ~ -1 +", paste(names(as.data.frame(cbind(X, Z))),
                                              collapse= "+")))
  x_vec <- colnames(X)
  index_i <- vector("list", length(levels(ID)))
  for(i in 1:length(index_i)){
    index_i[[i]] <- which(ID == levels(ID)[i])}
  b_i <- matrix(nrow = k, ncol = ncol(X), 0)
  colnames(b_i) <- x_vec
  dat <- as.data.frame(cbind(X, Z))
  dat_copy <- dat
  y_copy <- y
  for(i in 1:k){
    dat <- dat_copy
    y <- y_copy
    index <- NULL
    for(j in 1:length(index_i)){
      ho <- index_i[[j]]
      index <- c(index, sample(ho, size = length(ho), replace = T))
    }
    dat <- dat[index, ]
    y <- y[index]
    m_i <- rq(fmla, lambda = c(rep(0, ncol(X)), rep(lambda_min, ncol(Z))),
              method = "lasso", data = dat, tau = tau)
    b_i[i, ] <- m_i$coefficients[1:ncol(X)]
  }
  lower <- apply(b_i, MAR = 2, FUN = quantile, probs = c(0.025))
  upper <- apply(b_i, MAR = 2, FUN = quantile, probs = c(0.975))
  return(list(lower = lower, upper = upper))}
```

3.2.4 Detailed Results

Comparison methods are:

- rq without subject: `rq` from `quantreg` without Subject.
- rq with subject as fixed effect: `rq` from `quantreg` with Subject as fixed effects variable.

5%-Quantile Regression of Repeated Reaction-Times

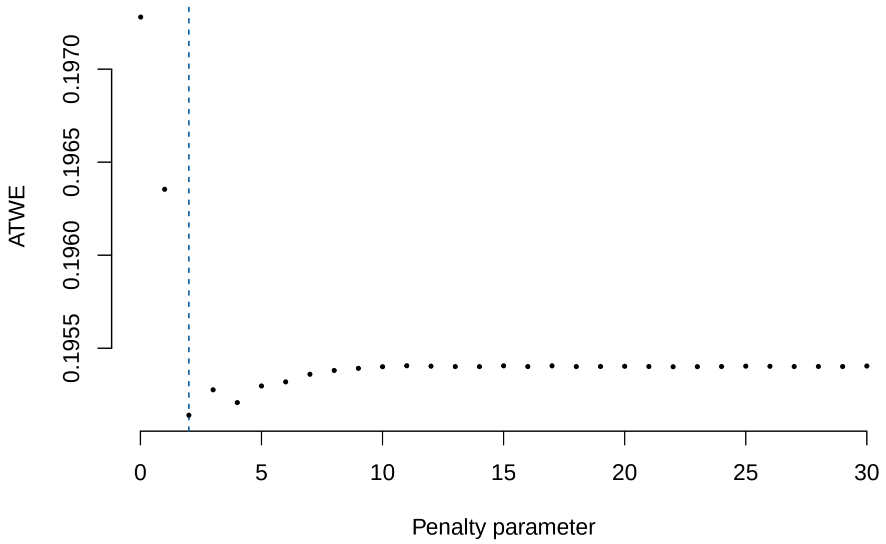

Figure S2: Average τ–weighted error (ATWE) across penalty parameter proposals.

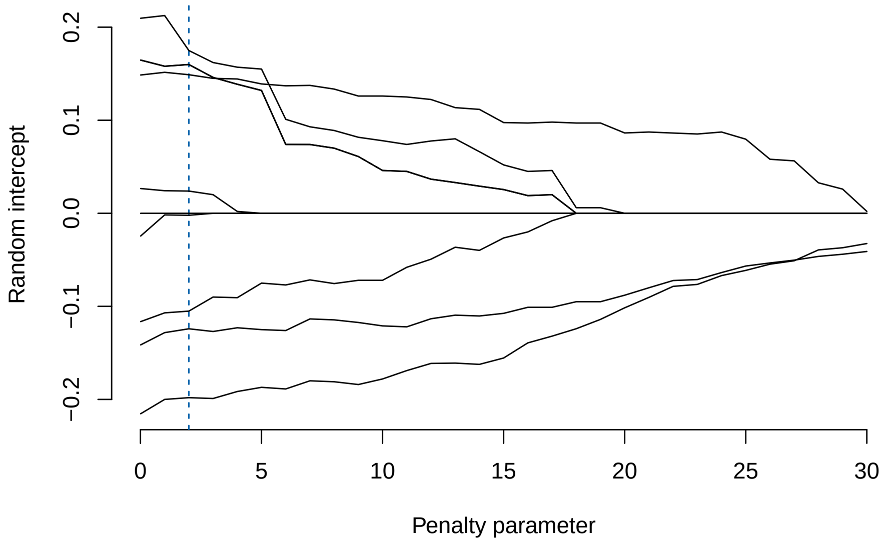

Figure S3: Normalized random intercepts across penalty parameter proposals.

	$\hat{\boldsymbol{\beta}}_{\texttt{rq}}$ without subject	$\hat{\boldsymbol{\beta}}_{\texttt{rq}}$ with subject as fixed effect	$\hat{\boldsymbol{\beta}}$	$\mathrm{CI}_{\mathrm{low}}$	$\mathrm{CI}_{\mathrm{up}}$
Intercept	0.899	0.959	0.938	0.823	1.052
PartnerP	0.042	0.063	0.068	-0.015	0.163
NoveltyO	0.007	0.014	0.017	-0.053	0.078
Block2	0.034	0.014	0.012	-0.054	0.076
DirectionW	0.017	-0.006	-0.010	-0.100	0.103
ExtremityM	-0.015	-0.025	-0.014	-0.101	0.078
IntDrcExt	0.005	0.043	0.052	-0.104	0.161
IntDrcPartner_P	-0.063	-0.088	-0.090	-0.223	0.046

10%-Quantile Regression of Repeated Reaction-Times

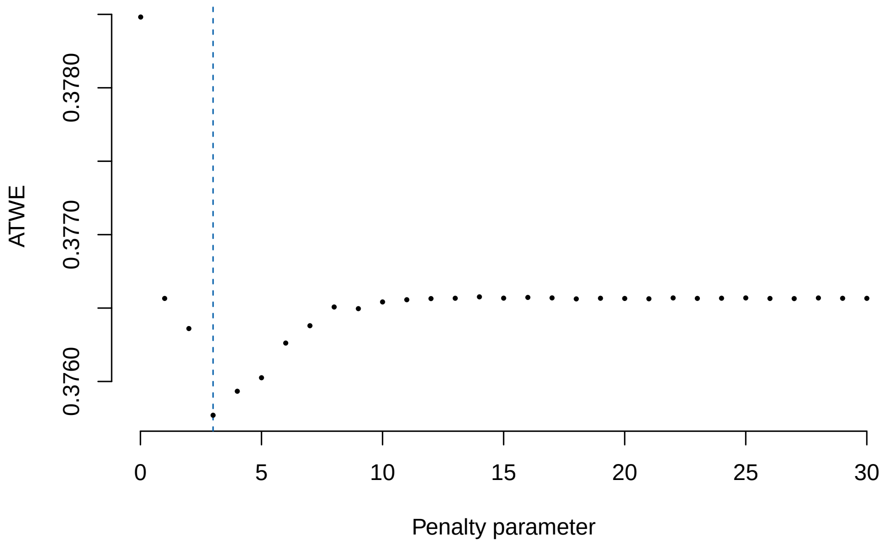

Figure S4: Average τ–weighted error (ATWE) across penalty parameter proposals.

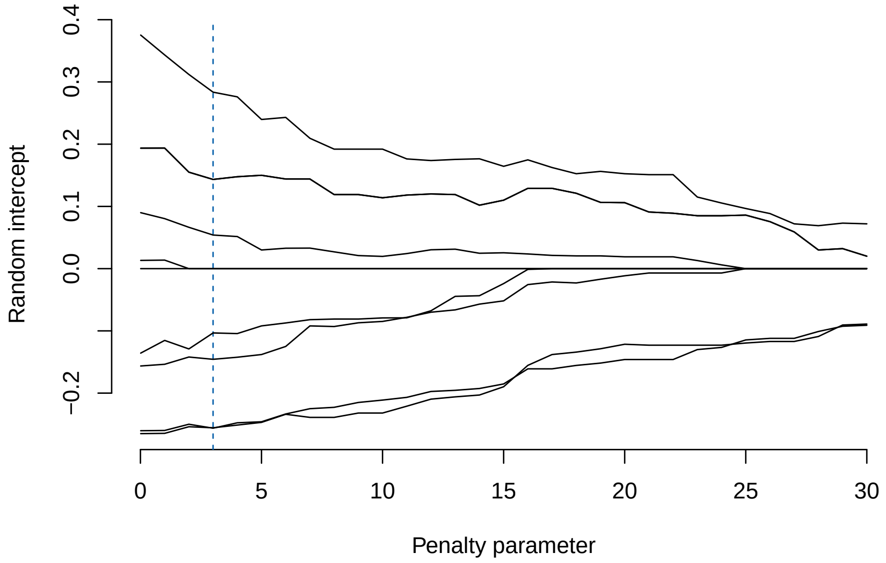

Figure S5: Normalized random intercepts across penalty parameter proposals.

	$\hat{\boldsymbol{\beta}}_{\texttt{rq}}$ without subject	$\hat{\boldsymbol{\beta}}_{\texttt{rq}}$ with subject as fixed effect	$\hat{\boldsymbol{\beta}}$	CI_{low}	CI_{up}
Intercept	1.104	1.133	1.156	0.997	1.243
PartnerP	0.020	0.079	0.085	-0.017	0.182
NoveltyO	-0.026	-0.004	-0.017	-0.091	0.058
Block2	0.016	-0.020	-0.023	-0.084	0.078
DirectionW	0.040	0.065	0.078	-0.067	0.177
ExtremityM	0.049	0.022	0.015	-0.080	0.107
IntDrcExt	-0.022	-0.019	-0.019	-0.157	0.127
IntDrcPartner_P	-0.090	-0.133	-0.146	-0.269	0.010

20%-Quantile Regression of Repeated Reaction-Times

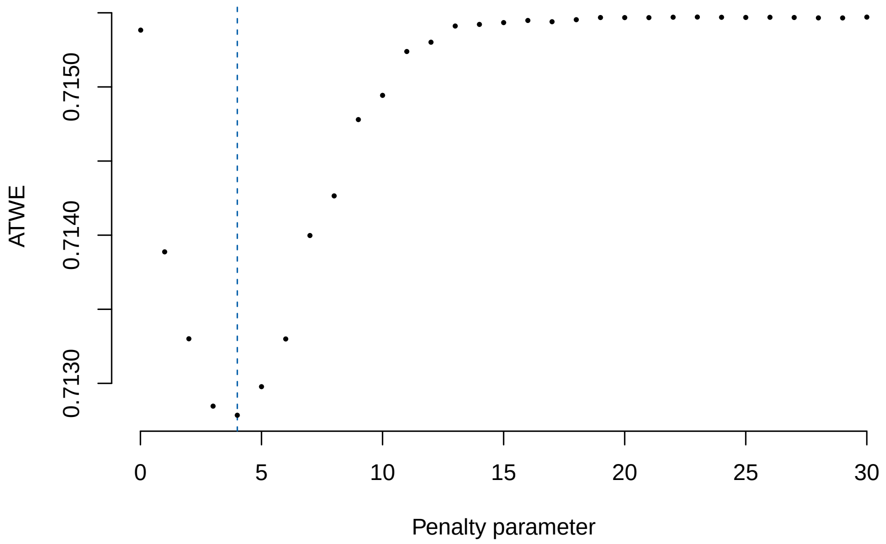

Figure S6: Average τ–weighted error (ATWE) across penalty parameter proposals.

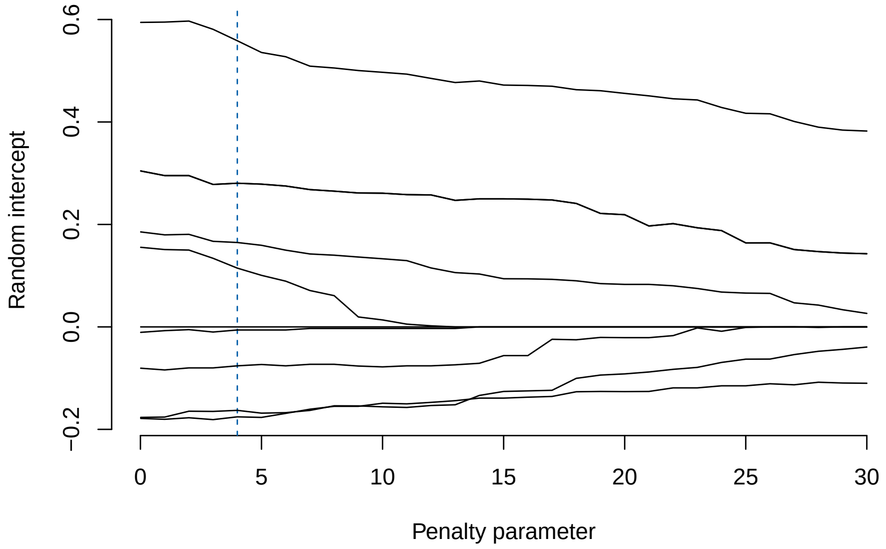

Figure S7: Normalized random intercepts across penalty parameter proposals.

	$\hat{\boldsymbol{\beta}}_{\texttt{rq}}$ without subject	$\hat{\boldsymbol{\beta}}_{\texttt{rq}}$ with subject as fixed effect	$\hat{\boldsymbol{\beta}}$	$\mathrm{CI}_{\mathrm{low}}$	$\mathrm{CI}_{\mathrm{up}}$
Intercept	1.339	1.407	1.342	1.213	1.474
PartnerP	0.057	0.081	0.069	-0.035	0.193
NoveltyO	-0.035	-0.033	-0.044	-0.120	0.053
Block2	0.016	-0.013	-0.009	-0.097	0.080
DirectionW	0.139	0.115	0.115	-0.032	0.243
ExtremityM	0.082	0.073	0.069	-0.049	0.174
IntDrcExt	-0.156	-0.094	-0.111	-0.253	0.095
IntDrcPartner_P	-0.125	-0.153	-0.150	-0.321	0.023

30%-Quantile Regression of Repeated Reaction-Times

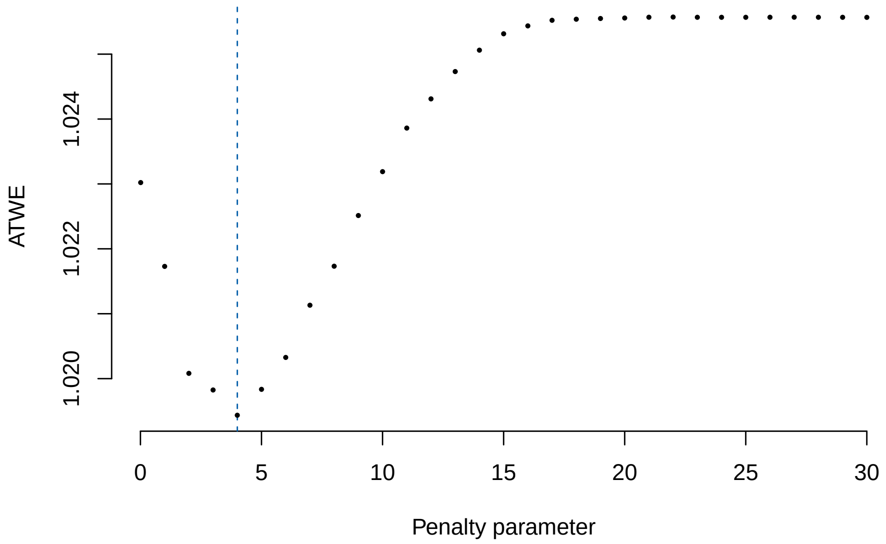

Figure S8: Average τ–weighted error (ATWE) across penalty parameter proposals.

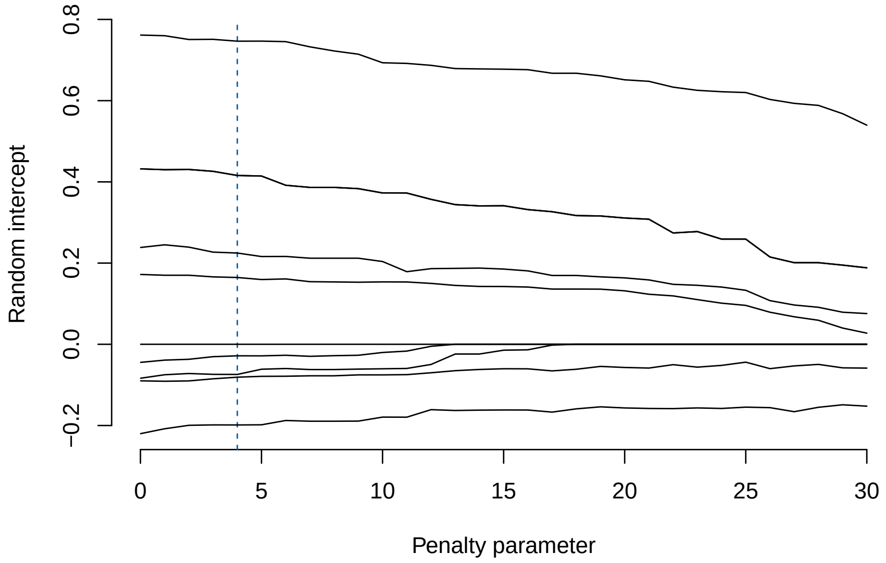

Figure S9: Normalized random intercepts across penalty parameter proposals.

	$\hat{\boldsymbol{\beta}}_{\texttt{rq}}$ without subject	$\hat{\boldsymbol{\beta}}_{\texttt{rq}}$ with subject as fixed effect	$\hat{\boldsymbol{\beta}}$	$\mathrm{CI}_{\mathrm{low}}$	$\mathrm{CI}_{\mathrm{up}}$
Intercept	1.613	1.610	1.476	1.374	1.709
PartnerP	0.096	0.113	0.121	-0.016	0.263
NoveltyO	-0.009	-0.025	-0.020	-0.117	0.079
Block2	-0.017	0.017	0.014	-0.088	0.114
DirectionW	0.169	0.233	0.241	0.024	0.370
ExtremityM	0.095	0.139	0.139	-0.029	0.245
IntDrcExt	-0.169	-0.191	-0.198	-0.353	0.036
IntDrcPartner_P	-0.182	-0.222	-0.240	-0.392	-0.012

40%-Quantile Regression of Repeated Reaction-Times

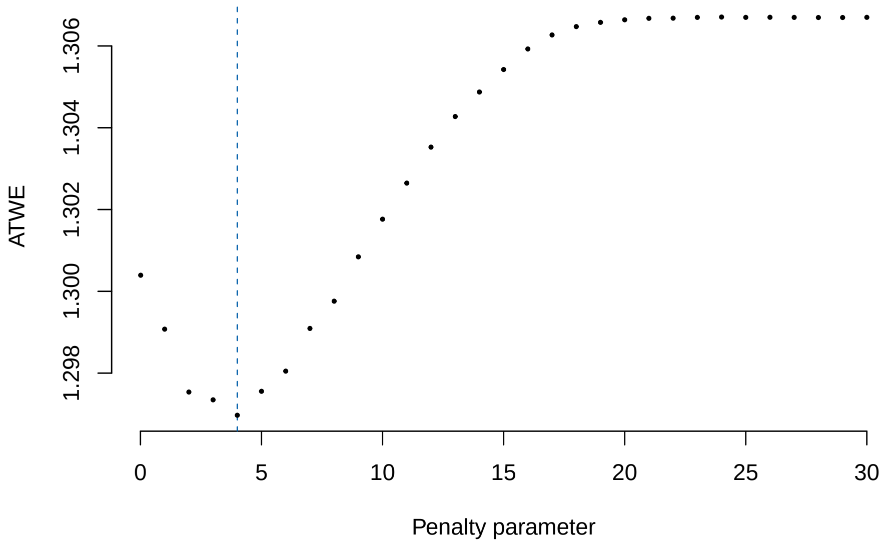

Figure S10: Average τ–weighted error (ATWE) across penalty parameter proposals.

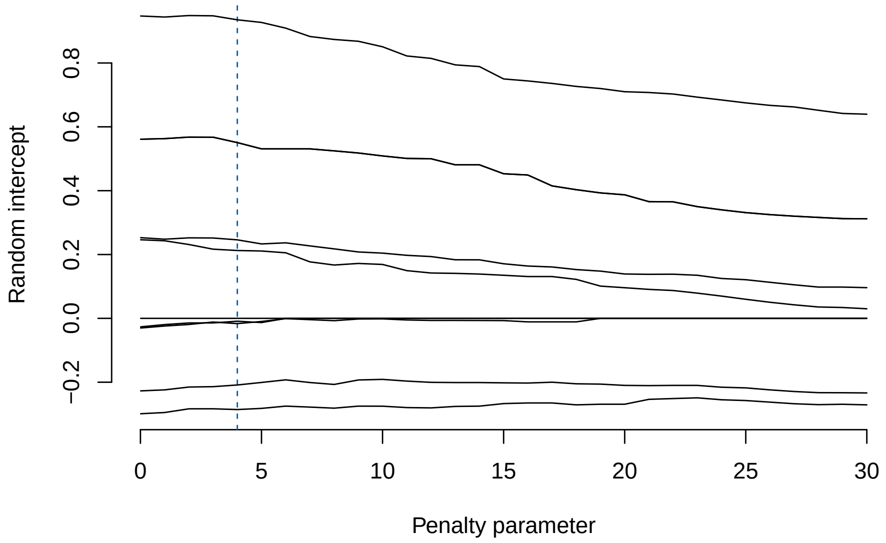

Figure S11: Normalized random intercepts across penalty parameter proposals.

	$\hat{\boldsymbol{\beta}}_{\texttt{rq}}$ without subject	$\hat{\boldsymbol{\beta}}_{\texttt{rq}}$ with subject as fixed effect	$\hat{\boldsymbol{\beta}}$	CI_{low}	CI_{up}
Intercept	1.823	1.913	1.762	1.618	2.018
PartnerP	0.144	0.127	0.118	-0.029	0.287
NoveltyO	0.024	0.012	0.018	-0.116	0.119
Block2	0.039	0.046	0.059	-0.060	0.171
DirectionW	0.229	0.205	0.181	-0.003	0.398
ExtremityM	0.129	0.117	0.101	-0.058	0.270
IntDrcExt	-0.148	-0.133	-0.122	-0.340	0.121
IntDrcPartner_P	-0.199	-0.210	-0.199	-0.436	0.017

Median Regression of Repeated Reaction-Times

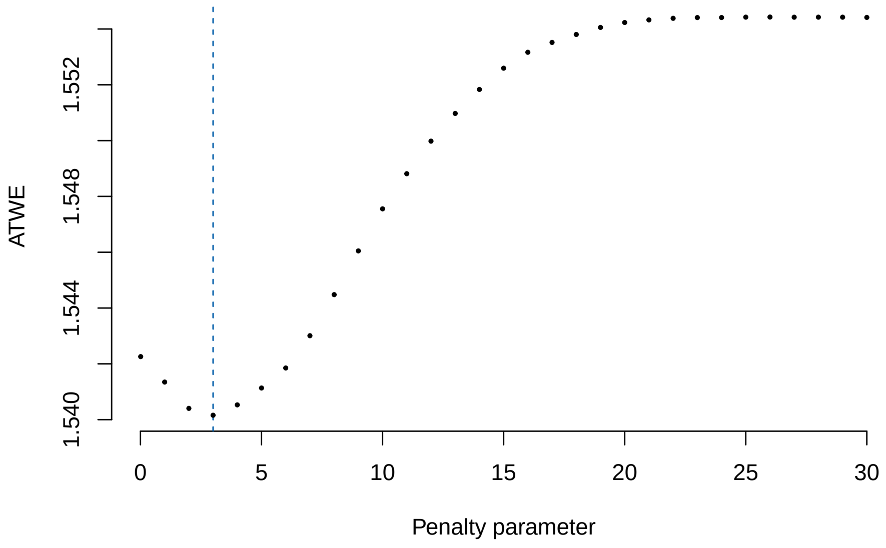

Figure S12: Average τ–weighted error (ATWE) across penalty parameter proposals.

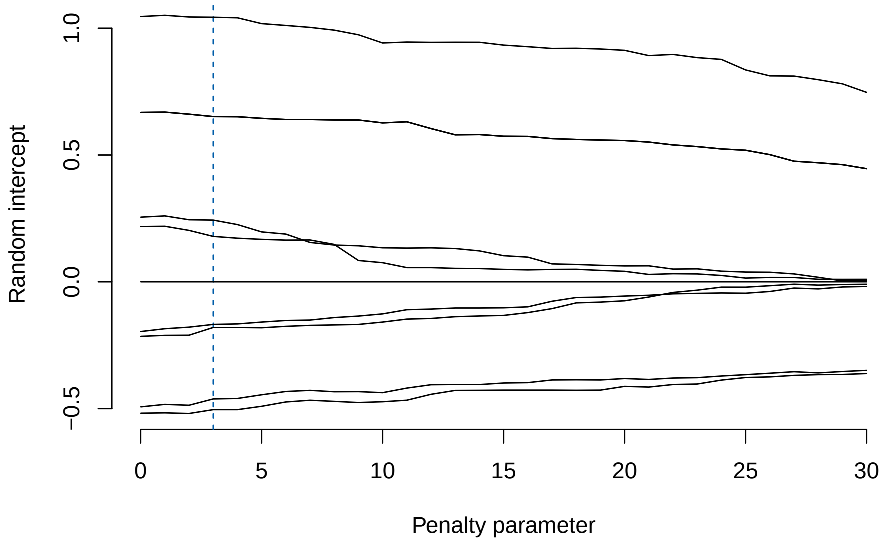

Figure S13: Normalized random intercepts across penalty parameter proposals.

	$\hat{\boldsymbol{\beta}}_{\texttt{rq}}$ without subject	$\hat{\boldsymbol{\beta}}_{\texttt{rq}}$ with subject as fixed effect	$\hat{\boldsymbol{\beta}}$	$\mathrm{CI}_{\mathrm{low}}$	$\mathrm{CI}_{\mathrm{up}}$
Intercept	2.221	2.303	2.197	1.932	2.481
PartnerP	0.118	0.068	0.085	-0.143	0.282
NoveltyO	0.026	0.004	0.026	-0.147	0.162
Block2	0.078	0.151	0.145	-0.016	0.297
DirectionW	0.175	0.117	0.125	-0.125	0.446
ExtremityM	0.141	0.108	0.093	-0.094	0.316
IntDrcExt	-0.122	-0.122	-0.092	-0.403	0.186
IntDrcPartner_P	-0.206	-0.115	-0.133	-0.447	0.172

60%-Quantile Regression of Repeated Reaction-Times

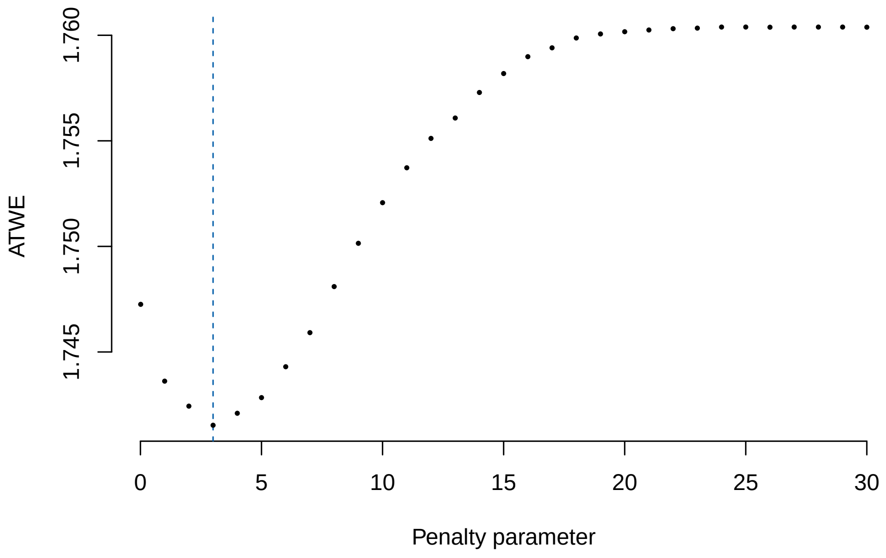

Figure S14: Average τ–weighted error (ATWE) across penalty parameter proposals.

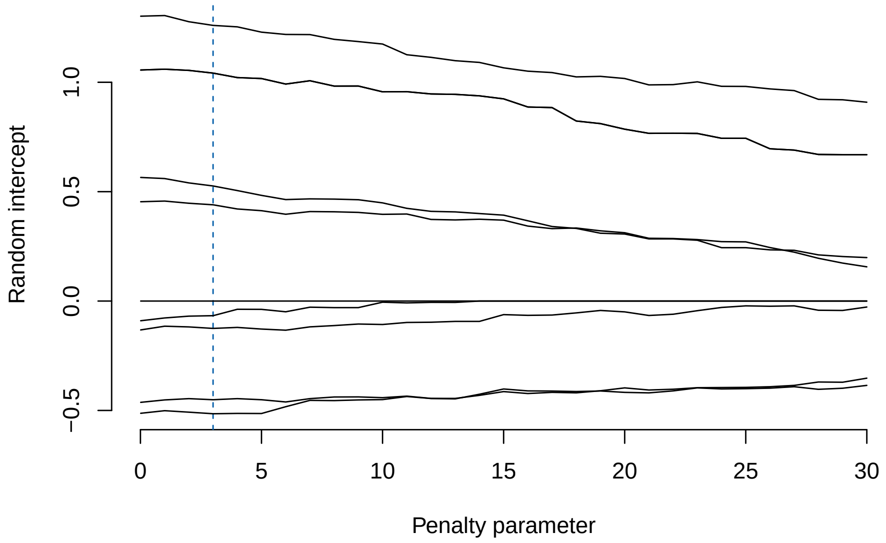

Figure S15: Normalized random intercepts across penalty parameter proposals.

	$\hat{\boldsymbol{\beta}}_{\texttt{rq}}$ without subject	$\hat{\boldsymbol{\beta}}_{\texttt{rq}}$ with subject as fixed effect	$\hat{\boldsymbol{\beta}}$	CI_{low}	CI_{up}
Intercept	2.645	2.728	2.516	2.253	2.939
PartnerP	0.059	0.078	0.093	-0.177	0.327
NoveltyO	0.099	0.020	0.030	-0.170	0.213
Block2	0.046	0.131	0.114	-0.059	0.320
DirectionW	0.208	0.255	0.225	-0.068	0.626
ExtremityM	0.184	0.130	0.099	-0.095	0.371
IntDrcExt	-0.057	-0.181	-0.130	-0.501	0.216
IntDrcPartner_P	-0.167	-0.190	-0.202	-0.602	0.158

70%-Quantile Regression of Repeated Reaction-Times

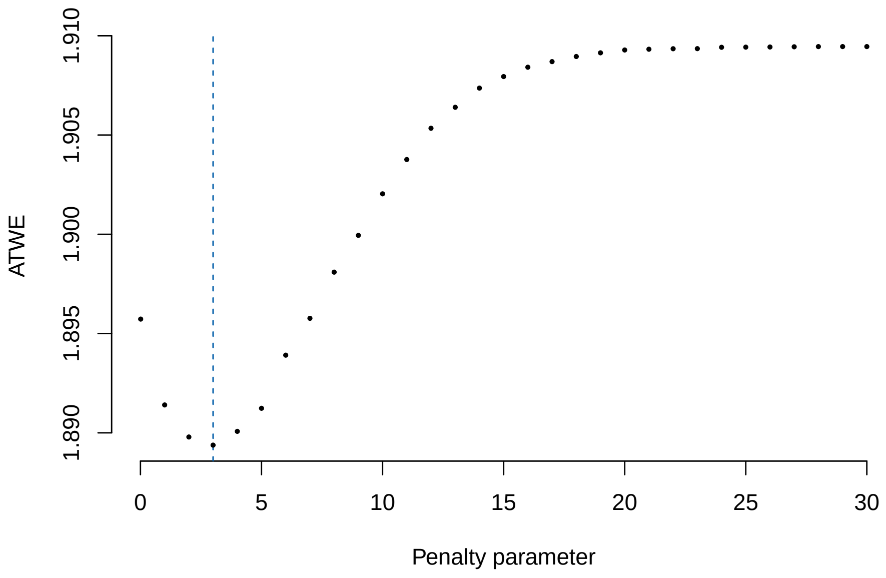

Figure S16: Average τ–weighted error (ATWE) across penalty parameter proposals.

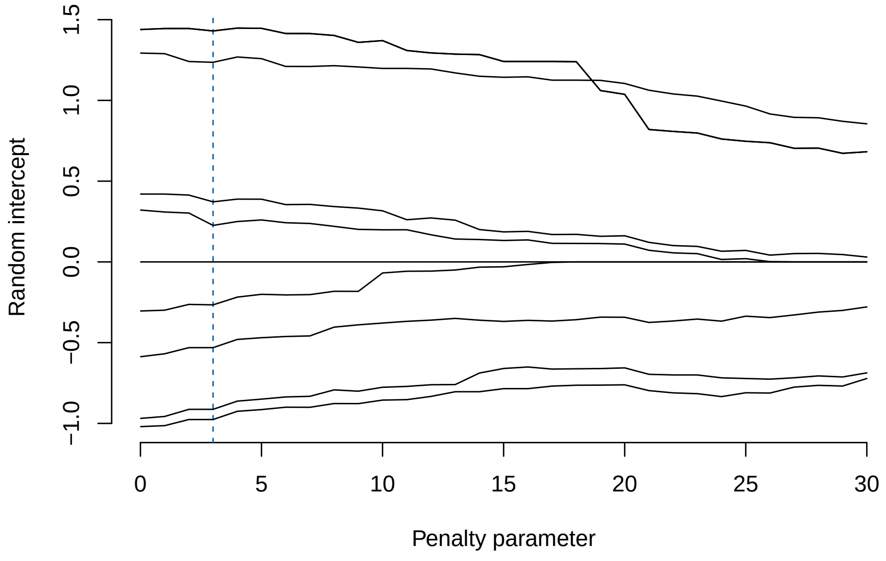

Figure S17: Normalized random intercepts across penalty parameter proposals.

	$\hat{\boldsymbol{\beta}}_{\texttt{rq}}$ without subject	$\hat{\boldsymbol{\beta}}_{\texttt{rq}}$ with subject as fixed effect	$\hat{\boldsymbol{\beta}}$	CI_{low}	CI_{up}
Intercept	3.200	3.293	3.209	2.735	3.738
PartnerP	0.252	0.193	0.212	-0.143	0.521
NoveltyO	0.096	0.065	0.047	-0.192	0.316
Block2	0.091	0.147	0.126	-0.120	0.404
DirectionW	0.254	0.536	0.527	0.007	0.952
ExtremityM	0.141	0.134	0.113	-0.220	0.471
IntDrcExt	0.145	-0.142	-0.112	-0.590	0.469
IntDrcPartner_P	-0.422	-0.565	-0.540	-1.038	-0.018

80%-Quantile Regression of Repeated Reaction-Times

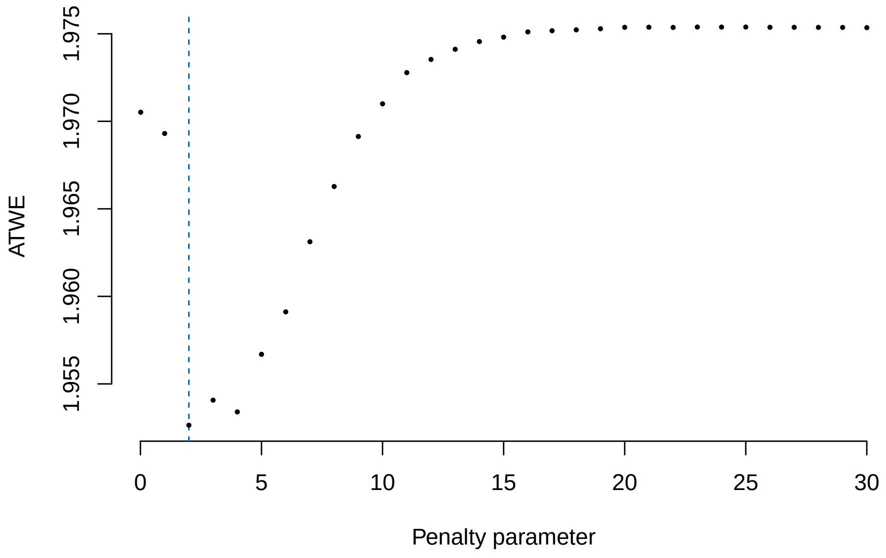

Figure S18: Average τ–weighted error (ATWE) across penalty parameter proposals.

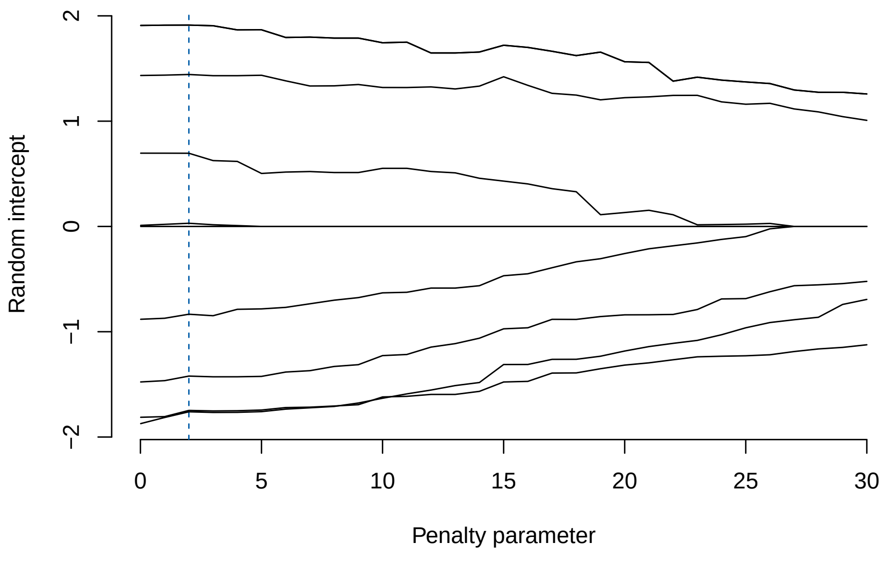

Figure S19: Normalized random intercepts across penalty parameter proposals.

	$\hat{\boldsymbol{\beta}}_{\texttt{rq}}$ without subject	$\hat{\boldsymbol{\beta}}_{\texttt{rq}}$ with subject as fixed effect	$\hat{\boldsymbol{\beta}}$	$\mathrm{CI}_{\mathrm{low}}$	$\mathrm{CI}_{\mathrm{up}}$
Intercept	4.109	4.343	4.507	3.621	5.029
PartnerP	0.683	0.270	0.268	-0.270	0.719
NoveltyO	0.201	0.246	0.290	-0.166	0.660
Block2	-0.054	0.213	0.217	-0.131	0.635
DirectionW	0.625	0.779	0.787	0.098	1.422
ExtremityM	0.118	-0.024	-0.039	-0.424	0.554
IntDrcExt	0.424	0.220	0.211	-0.621	0.919
IntDrcPartner_P	-1.100	-0.963	-0.941	-1.675	-0.142

90%-Quantile Regression of Repeated Reaction-Times

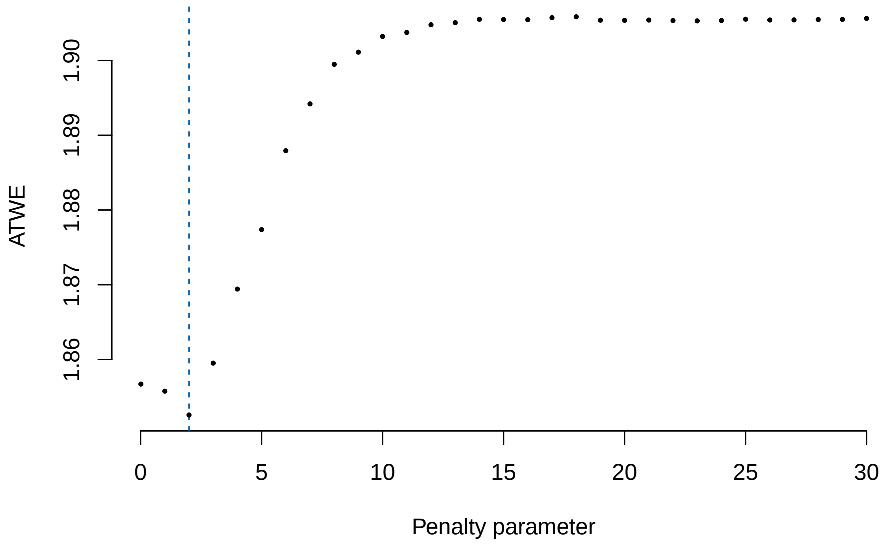

Figure S20: Average τ–weighted error (ATWE) across penalty parameter proposals.

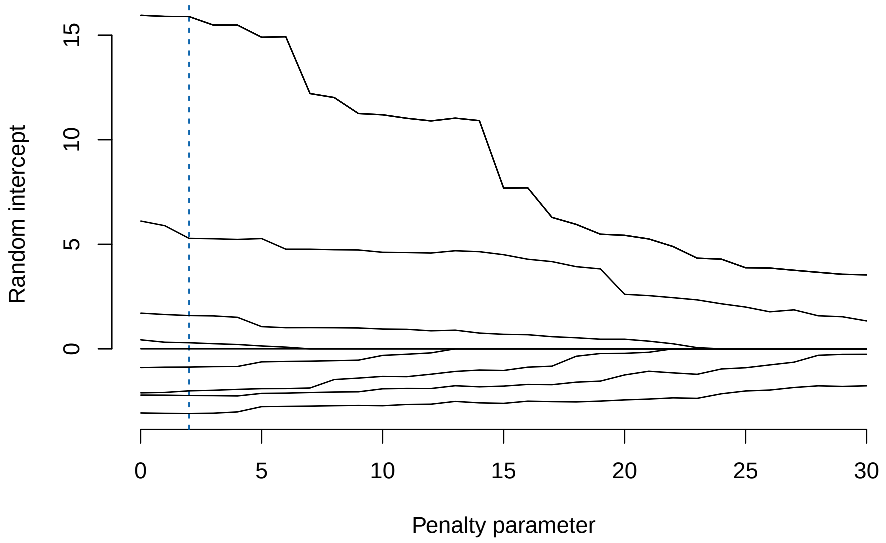

Figure S21: Normalized random intercepts across penalty parameter proposals.

	$\hat{\boldsymbol{\beta}}_{\mathtt{rq}}$ without subject	$\hat{\boldsymbol{\beta}}_{\mathtt{rq}}$ with subject as fixed effect	$\hat{\boldsymbol{\beta}}$	$\mathrm{CI}_{\mathrm{low}}$	$\mathrm{CI}_{\mathrm{up}}$
Intercept	6.129	7.791	6.046	4.783	7.641
PartnerP	0.542	0.144	0.163	-0.952	1.260
NoveltyO	0.740	0.417	0.327	-0.347	1.634
Block2	0.122	0.357	0.379	-0.823	0.997
DirectionW	4.450	3.494	3.728	0.599	5.835
ExtremityM	-0.247	0.790	0.767	-0.615	1.632
IntDrcExt	0.662	-0.290	-0.293	-2.126	2.055
IntDrcPartner_P	-4.620	-3.574	-3.768	-5.940	-0.695

95%-Quantile Regression of Repeated Reaction-Times

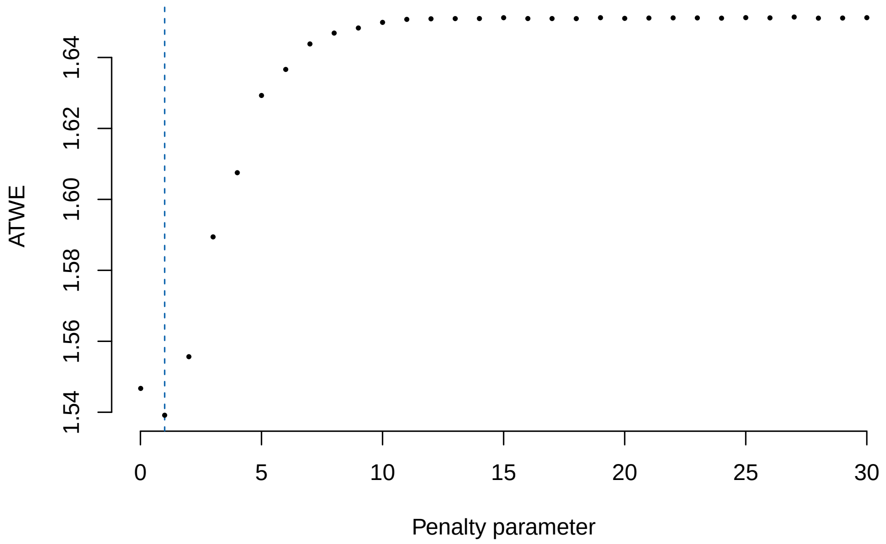

Figure S22: Average τ–weighted error (ATWE) across penalty parameter proposals.

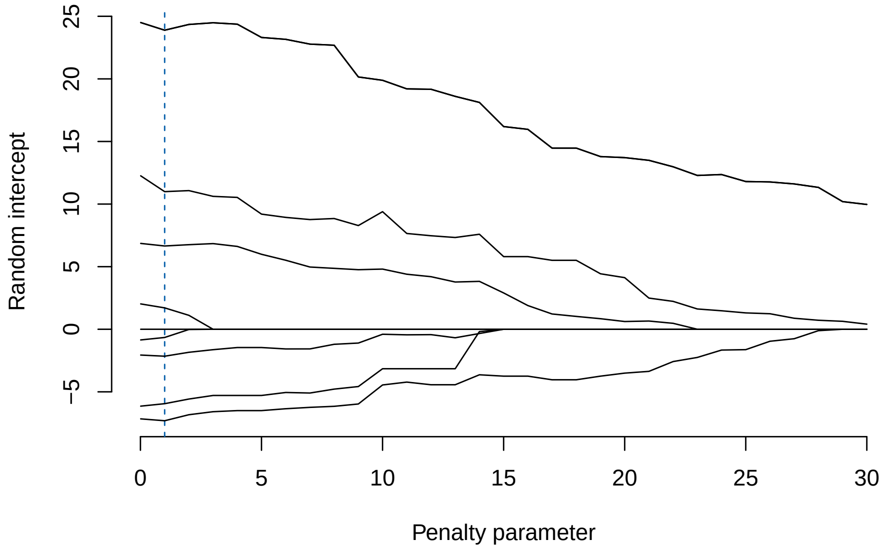

Figure S23: Normalized random intercepts across penalty parameter proposals.

	$\hat{\boldsymbol{\beta}}_{\texttt{rq}}$ without subject	$\hat{\boldsymbol{\beta}}_{\texttt{rq}}$ with subject as fixed effect	$\hat{\boldsymbol{\beta}}$	CI_{low}	CI_{up}
Intercept	10.523	14.889	11.824	6.639	14.299
PartnerP	3.991	0.315	0.773	-1.454	5.590
NoveltyO	3.178	0.916	0.568	-0.788	3.619
Block2	0.258	-0.750	-0.693	-2.515	1.294
DirectionW	11.666	6.780	7.352	2.751	12.794
ExtremityM	0.875	1.029	0.760	-1.302	5.298
IntDrcExt	-2.808	-1.253	-1.387	-6.221	3.603
IntDrcPartner_P	-14.715	-7.416	-8.099	-14.301	-3.097

3.3 Verification Using Bayesian Quantile Regression of Repeated Reaction-Times

To verify our findings from the frequentist Quantile Regression framework, we applied a Bayesian Quantile Regression framework to the reaction-times with Ridge-penalization priors on the nuisance parameters capturing subject-specific heterogeneity. The Bayesian formulation of Quantile Regression relies on assuming the asymmetric Laplace distribution as auxiliary error distribution, yielding posterior modes in analogy to frequentist estimates. This approach is implemented in BayesX (Brezger et al., 2005) using Markov chain Monte Carlo (MCMC) sampling from the posterior distribution, and described in Waldmann et al. (2013). The results are shown in Figure S24: We only get slight differences in the coefficient estimates and, in conclusion, this reaffirms our findings on the upper quantiles of reaction-time.

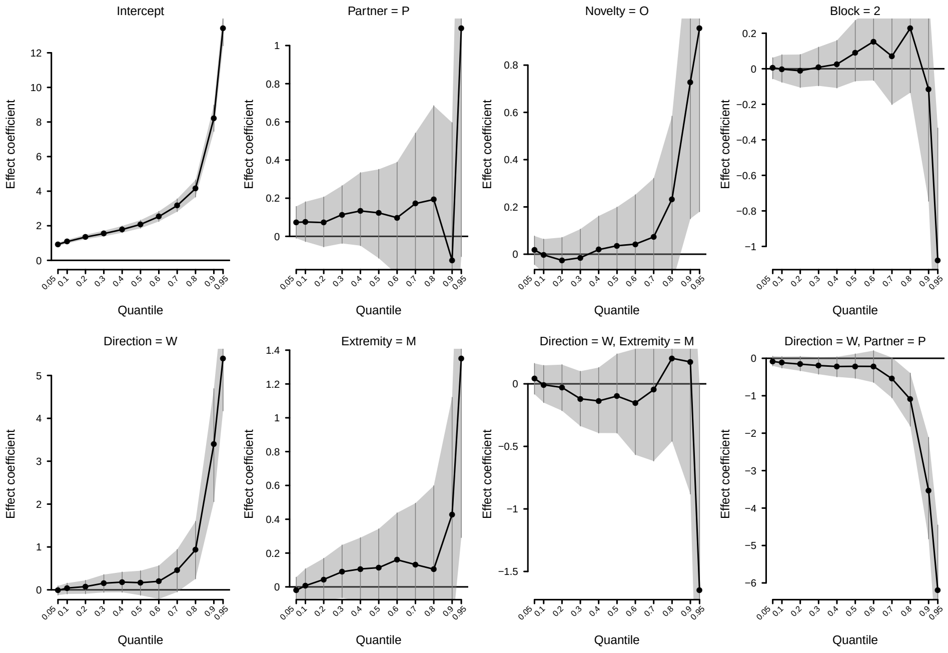

Figure S24: Results from Bayesion Quantile Regression.

References

D. Bates, M. Mächler, B. Bolker, and S. Walker. Fitting linear mixed-effects models using lme4. *Journal of Statistical Software*, 67(1):1–48, 2015. doi: 10.18637/jss.v067.i01.

A. Brezger, T. Kneib, and S. Lang. Bayesx: Analyzing bayesian structural additive regression models. *Journal of Statistical Software*, 14(1):1–22, 2005. ISSN 1548-7660. doi: 10.18637/jss.v014.i11.

H. Haupt, K. Kagerer, and J. Schnurbus. Cross-validating fit and predictive accuracy of nonlinear quantile regressions. *Journal of Applied Statistics*, 38(12):2939–2954, 2011. doi: 10.1080/02664763.2011.573542.

A. Karlsson. Bootstrap methods for bias correction and confidence interval estimation for nonlinear quantile regression of longitudinal data. *Journal of Statistical Computation and Simulation*, 79(10):1205–1218, 2009. doi: 10.1080/00949650802221180.

R. Koenker. Quantile regression for longitudinal data. *Journal of Multivariate Analysis*, 91(2):74–89, 2004. doi: 10.1016/j.jmva.2004.05.006.

R. Koenker. *quantreg: Quantile Regression*, 2015. URL `http://CRAN.R-project.org/package=quantreg`. R package version 5.19.

R Core Team. *R: A Language and Environment for Statistical Computing*. R Foundation for Statistical Computing, Vienna, Austria, 2015. URL `https://www.R-project.org/`.

E. Waldmann, T. Kneib, Y. Yue, S. Lang, and C. Flexeder. Bayesian semiparametric additive quantile regression. *Statistical Modelling*, 13:223–252, 2013.
